# A Repertoire of Clinical Non-Dermatophytes Moulds

**DOI:** 10.3390/jof9040433

**Published:** 2023-03-31

**Authors:** Estelle Menu, Quentin Filori, Jean-Charles Dufour, Stéphane Ranque, Coralie L’Ollivier

**Affiliations:** 1 Laboratoire de Parasitologie-Mycologie, IHU Méditerranée Infection, 13385 Marseille, France; 2 Institut de Recherche pour le Développement, Assistance Publique-Hôpitaux de Marseille, Service de Santé des Armées, VITROME: Vecteurs-Infections Tropicales et Méditerra-néennes, Aix Marseille Université, 13385 Marseille, France; 3 INSERM, IRD, SESSTIM, Sciences Economiques & Sociales de la Santé & Traitement de l’Information Médicale, ISSPAM, Aix Marseille University, 13385 Marseille, France; 4 APHM, Hôpital de la Timone, Service Biostatistique et Technologies de l’Information et de la Communication, 13385 Marseille, France

**Keywords:** mould, filamentous fungi, repertoire

## Abstract

Humans are constantly exposed to micromycetes, especially filamentous fungi that are ubiquitous in the environment. In the presence of risk factors, mostly related to an alteration of immunity, the non-dermatophyte fungi can then become opportunistic pathogens, causing superficial, deep or disseminated infections. With new molecular tools applied to medical mycology and revisions in taxonomy, the number of fungi described in humans is rising. Some rare species are emerging, and others more frequent are increasing. The aim of this review is to (i) inventory the filamentous fungi found in humans and (ii) provide details on the anatomical sites where they have been identified and the semiology of infections. Among the 239,890 fungi taxa and corresponding synonyms, if any, retrieved from the Mycobank and NCBI Taxonomy databases, we were able to identify 565 moulds in humans. These filamentous fungi were identified in one or more anatomical sites. From a clinical point of view, this review allows us to realize that some uncommon fungi isolated in non-sterile sites may be involved in invasive infections. It may present a first step in the understanding of the pathogenicity of filamentous fungi and the interpretation of the results obtained with the new molecular diagnostic tools.

## 1. Introduction

It is estimated that there are between 1.5 and 5 million fungal species on Earth, and about 100,000 species are currently described [1,2]. Of these species, only a few hundred have the capacity to infect humans [3]. Humans are constantly exposed to potential fungal pathogens, as they are part of their normal flora and that of soil, water and air [3]. Moulds are a part of the vast kingdom of fungi alongside yeasts, mushrooms, polypores, plant parasitic rusts and smuts, microsporidia and *Pneumocystis*. Filamentous fungi are ubiquitous in the environment and can lead to opportunistic diseases presenting as superficial, invasive or disseminated infections. The number of described species is constantly increasing, probably due to the popularisation of DNA-based diagnostic tools, which now allow the distinction between close taxa and the identification of fungi, even in small quantities [1,4]. The taxonomy of fungi is also in constant evolution with the “one fungus, one name” nomenclature and integrative taxonomy approach combining genomics, morphology and ecology [1,5]. In recent years, epidemiological changes in invasive fungal diseases have been observed, new risk factors have emerged, and the number of patients at risk of developing these infections is also increasing [6,7]. Medical mycology is, therefore, a constantly evolving dynamic. *Aspergillus*, *Penicillium*, mucorales and dematiaceous fungi are the main filamentous fungi taxa involved in human diseases. Current reviews mainly focused on these taxa [3,8,9]. However, other, rarer species of moulds can emerge in specific infection sites, such as *Paecilomyces variotii* or *Purpureocillium lilacinum* in sino-pulmonary fungal infections, and should not be overlooked [10].

In this review, we offer an overview, as of 16 June 2020, of the filamentous fungi identified in humans by culture and nucleotide analyses associated or not with histopathology. We also provide information on the organs where these micromycetes are isolated and on the semiology of the infections. We have chosen to divide our review into two approaches. First, we describe the taxa of interest and indicate their preferred site of infection. We then described which filamentous fungi were involved at each major anatomical site.

## 2. Materials and Methods

### 2.1. Systematic Literature Review and Database Creation

**Figure 1:**
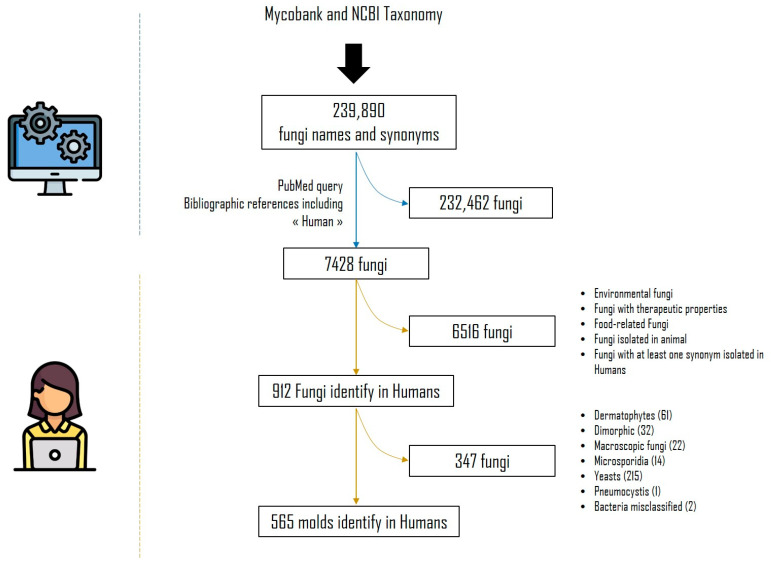
Systematic literature review flowchart.

First, all fungi names and synonyms were collected on both Mycobank (https://www.mycobank.org/, accessed on 15 November 2019) and NCBI Taxonomy (https://www.ncbi.nlm.nih.gov/taxonomy, accessed on 15 November 2019) that represent the state of the art of taxonomy of microfungi, updated on 15 November 2019. From Mycobank, the downloaded fungi taxon names and synonyms were provided in the worksheet https://www.MycoBank.org/images/MBList.zip accessed on 15 November 2019. From NCBI Taxonomy, the query used was Fungi[subtree] AND species[rank] AND specified[prop]. Python script using the Biopython package [11] was also implemented to fetch synonyms from NCBI Taxonomy. We have aggregated and deduplicated these two fungi name listings in order to obtain a single list of 239,890 fungi taxa and corresponding synonyms, if any (Figure 1). For each fungus name in the list, we used a Python script and Biopython package [11] to query PubMed to find bibliographic references that mention the fungi name or its synonyms associated with the term “human” in the article title (TI), abstract (AB), author-supplied keywords (Other Term (OT)) or the Medical Subject Headings' (MeSH) terms. The syntax of the queries was dynamically built using this pattern (fungi_name_or_synonyms [TIAB] OR fungi_name_or_synonyms[OT] OR fungi_name_or_synonyms[MeSH]) AND (“Human”[TIAB] OR “Human”[OT] OR “Humans”[MeSH]). Based on the query performed on 15 November 2019, 7428 fungi taxa were found with at least one PubMed reference.

An MS Access^®^ database (Access 2013, Microsoft) was set up on 16 June 2020, with these 7428 recorded fungi names. Using this relational database management system, a link for each taxa corresponding to the query described above gave access to the relevant PubMed references and made it possible to study them one by one in order to identify and collect the relevant information.

### 2.2. Manual Database Incrementation

In the MS Access database (MS Access 2013 TM, Microsoft), each of the 7428 fungi had a record from a page linked to the PubMed-relevant references. For each PubMed reference, an analysis of the title and/or abstract and/or whole paper was performed manually to ensure that it was isolated from humans. This process was time-consuming. Only references present in PubMed before 16 June 2020 were taken into account in order to have the same PubMed content for each fungal species.

After analysis, 6516 fungal taxa that were ultimately not found in humans were excluded. They included fungi of food, therapeutic or environmental interest, or those involved in domesticated animal diseases. Synonyms, when not isolated from humans but associated with a species involved in humans, were also excluded. We focused on non-dermatophytes moulds. In fact, yeasts, microsporidia, dimorphic fungi, dermatophytes and *Pneumocystis* isolated in humans were also excluded.

We analysed the titles and/or abstracts and/or full paper and/or supplementary data, when available, of 565 moulds fungal names and synonyms isolated in humans to complete information on the anatomical site involved and the semiologies of the associated infection by filling in the PubMed Unique Identifier (PMID) of the publication concerned. Only identifications by direct diagnosis were taken into account, including culture (followed by morphological, Matrix-Assisted Laser Desorption Ionization Time of Flight (MALDI-TOF) mass spectrometry or DNA sequence-based identification) associated or not with histopathological findings and Polymerase Chain Reaction (PCR). Publications reporting a species-level diagnosis based solely on histopathological examination or indirect methods results were excluded. The date of first publication, last publication and the name used was also reported in the software.

The anatomical sites included: systemic (isolated from blood, bone marrow, blood vessels/arteries or lymph nodes), central nervous system (isolated from cerebrospinal fluid or brain biopsy); ophthalmic systems (isolated from ocular samples, such as vitreous humour, corneal scrapings or lacrimal fluid); heart (isolated from cardiac specimens, e.g., valve or pericardial fluid); osteo-articular system (isolated from joint or bone samples); skeletal muscles (isolated from muscles); soft-tissue (isolated from soft-tissue); endocrine glands (isolated from adrenal, pituitary gland or thyroid); skin system (isolated from cutaneous or subcutaneous samples); otorhinolaryngeal sphere (isolated from nasal specimens, including sinuses, and throat specimens, including mouth, tongue, oesophagus, larynx, pharynx or trachea); auditory system (isolated from ear samples); dental (isolated from tooth root, dental pulp or anatomical structures directly in contact with the tooth in case of periodontitis, gingivitis, implant infection or abscess); pulmonary (isolated from the upper respiratory tract, e.g., sputum, lower respiratory tract, bronchoalveolar lavage fluid, lung biopsy, bronchial brushing, bronchoscopic needle aspiration and bronchial aspirate, pleural fluid and mediastinal specimen); breast; urinary tract (isolated from the urinary tract including kidneys, ureters, bladder, urinary meatus and the prostate); genital (isolated from genitalia or related body fluids, both male and female, including penile and urethral samples); digestive system (isolated from stool samples or organs of the digestive system, including peritoneum, intestines, pancreas, spleen, gallbladder or appendix, but excluding the liver); hepatic (liver biopsy); and pregnancy (isolated from the placenta or foetus). When information on the anatomical site was provided, we added a degree of accuracy by specifying anatomical details or semiology of the associated infection (e.g., endophthalmitis for ocular sphere) by filling in the PMIDs. In brief, for each fungus with publications reporting having been identified in humans, the current name and dates of the first and last publications were completed by filling in a list of PMID for each anatomical site and the semiology of the associated infection.

### 2.3. Data Analysis

The MS Access^®^ database (Access 2013, Microsoft) was converted into two Excel files (Excel 2013, Microsoft). In the first file, the number of PMIDs per taxa was calculated by the anatomical site where the fungi were isolated. In the second, the number of PMIDs per fungus was given for six major fungal categories (i.e., *Aspergillus*, dematiaceous, *Fusarium*, mucorales, *Penicillium* and *Pseudallescheria*/*Scedosporium*) according to the infections associated with the isolation of the fungus, as stated by the authors of the article. If more than one case was described in a publication with the same anatomical site, it counted as one publication because of a single PMID.

### 2.4. Taxonomy

Taxa were organised at the high-level classification into sections or species complexes based on https://www.aspergilluspenicillium.org/ (accessed on 1 September 2022) for *Aspergillus* spp. [12] and *Penicillium* spp. [13,14] and relevant publications for *Fusarium* spp. [15,16]. Mucorales and dematiaceous fungi were classified by genus [17,18].

### 2.5. Synonyms

We referred to the “current name” in Mycobank to identify the current name and the synonyms. The current name/synonym association was then checked by querying the PubMed database.

### 2.6. Figures

All figures were produced using the online tool, Wordart (https://wordart.com/). The size of the name of each species was proportional to the number of times it occurred in the database. The genus *Aspergillus* is represented in red, the dematiaceous fungi in brown, the mucorales in blue, the genus *Fusarium* in turquoise, the *Scedosporium*/*Lometospora* species complex in purple, the genus *Penicillium* in green and the others in black.

## 3. Results

### 3.1. Fungal Location by Focusing on the Predominant Genus

In total, 6913 articles/PMIDs were included. This bibliographical research identified 565 fungal species of 192 genera, which had been reported at least once in humans. The list of theses taxa is detailed in Table 1, with their former and current scientific name, if applicable. Briefly, there were 204 dematiaceous fungi, 81 *Aspergillus* spp., 25 *Penicillium* spp., 35 *Fusarium* spp., 45 mucorales, 14 of to the *Scedosporium*/*Lomentospora* complex, and 161 to other mould species distributed in 103 genera. The results obtained for each of these taxa will be presented below. Regarding the publications reporting the isolation of these micromycetes at all anatomical sites (a publication can be counted multiple times due to the possible report of multiple anatomical sites in the same publication), the leading genus was *Aspergillus* (total: 4401). The *Fumigati* and *Flavi* sections were the most recorded into this genus (total: 2671 and 865, respectively). In second place come the dematiaceous fungi (total: 1976), followed by the *Scedosporium*/*Lomentospora* complex (total: 1222), mucorales (total: 1089) and *Fusarium* (total: 713). *Penicillium* was rarely reported in human infections (total: 163).

#### 3.1.1. *Aspergillus*

*Aspergillus* species were the most frequent moulds isolated in human clinical samples. In this repertoire, the 81 *Aspergillus* species identified at least once in humans belong to 14 sections of the 20 described [19], as follows: *Aspergillus*, *Candidi*, *Circumdati*, *Clavati*, *Cremei*, *Flavi*, *Flavipedes*, *Fumigati*, *Nidulantes*, *Nigri*, *Polypaecilum*, *Restricti*, *Terrei* and *Usti*. As reported in the literature, the filamentous fungus mostly isolated from humans is *Aspergillus fumigatus* [20], followed by *Aspergillus flavus*, *Aspergillus niger* and *Aspergillus terreus*. The lung and respiratory tracts were the most common anatomical sites of infection, with a total of 1180 publications for *A. fumigatus*, 174 for *A. flavus*, 102 for *A. niger* and 81 publications for *A. terreus*. These fungi are indeed ubiquitous in the environment and are transmitted by the airways [21]. The results will be approached in terms of the *Aspergillus* section to rule out potential misidentifications related to morphological identification [4]. The anatomical site in the second position and concerning all the sections was the skin system. Cutaneous aspergillosis can be primary and can affect immunocompetent patients or can be secondary in cases of disseminated infection, predominantly in immunosuppressed patients [22,23,24]. The *Nigri* section has a tropism for the auditory system, with 79 publications, which has already been noted in the literature [25]. Regarding the clinical presentations involving the central nervous system or heart, the *Fumigati* section was predominantly represented (194 and 118 publications, respectively). The *Fumigati*, *Flavi* and *Nigri* sections had a predominantly ocular involvement, with 140, 93, and 36 publications, respectively.

#### 3.1.2. *Fusarium*

The genus *Fusarium* includes at least 200 species, grouped into about ten phylogenetic species complexes [15,16,26]. In this review of the literature, only eight species complexes were found to have been isolated from humans: *F. chlamydosporum* species complex (FCSC), *F. dimerum* species complex (FDSC), *F. incarnatum–F. equiseti* species complex (FIESC), *F. oxysporum* species complex (FOSC), *F. sambucinum* species complex (FSAMSC), *F. solani* species complex (FSSC), *F. fujikuroi* species complex (FFSC) and *Gibberella fujikuroi* species complex (GFSC). Interestingly, looking at the *Fusarium* genus as a whole, three anatomical sites stand out: the ocular system (190 publications), the cutaneous system (233 publications) and systemic involvement (134 publications). This is consistent with the data in the literature reporting superficial cases, mainly keratitis and onychomycosis in immunocompromised or immunocompromised patients and disseminated infections in immunocompromised patients [27,28,29,30]. Species belonging to the FSSC are predominantly represented, which has previously been shown to be the most virulent *Fusarium* species complex in animal models [31].

#### 3.1.3. *Penicillium*

The genus *Penicillium* is ubiquitous in the environment and is rarely involved in human infections. When found in superficial and aerial samples, they are often considered contaminants. In this literature review, only 163 publications reporting the isolation of these hyaline hyphomycetes in humans were found. One species has emerged since the 1990s and mainly affects humans with acquired immunodeficiency syndrome (AIDS): *Talaromyces marneffei* [32,33], and this is the predominant species within the genus *Penicillium* representing 54% of the publications (89/164). The members of the genus *Penicillium* are mostly reported in three anatomical sites: the pulmonary sphere (48 publications), the cutaneous system (23 publications) and systemic involvement (36 publications).

#### 3.1.4. Mucorales

The order of the mucorales (previously Zygomyces) includes the hyaline pauciseptated filamentous fungi group, comprising 13 genera: *Mucor*, *Lichtheimia*, *Rhizomucor*, *Rhizopus*, *Absidia*, *Syncephalastrum*, *Cunninghamella*, *Apophysomyces*, *Mycocladus*, *Saksenaea*, *Actinomucor*, *Thamnostylum* and *Thermomucor*. All of these are implicated in human infections [34]. In this literature review, *Rhizopus* was the genus mostly involved in human infections, with *Rhizopus oryzae* (121 publications) (plus its synonym and current appellation *Rhizopus arrhizus* (53 publications)) leading the way, followed by *Rhizopus microsporus* (90 publications). Mucorales can be classified according to the primary route of infection: airborne, direct contact with contaminated devices or by trauma [34,35]. Here, we compare the number of publications reporting isolation in the skin system versus the respiratory system, including from the oto-rhino-laryngological sphere and pulmonary sphere: The genera *Apophysomyces* (54 versus 26 publications, respectively), *Mucor* (40 versus 18 publications, respectively) and *Saksenaea* (45 versus eight publications, respectively) are mostly isolated from the skin system and therefore transmitted by contact; the genera *Cunninghamella* (12 versus 52 publications, respectively), *Rhizopus* (75 versus 134 publications, respectively) and *Rhizomucor* (8 versus 31 publications, respectively) are mostly isolated from the oto-rhino-laryngological sphere and pulmonary system and are, therefore, airborne; finally, *Lichtheimia* (77 versus 74 publications, respectively) had a less obvious tropism for one or the other of these two systems. The cutaneous or respiratory tropism can be explained by the differences found in the sporangia of these genera [35]. In fact, the wet spores of the *Mucor*, *Apophysomyces* and *Saksenaea* species are probably not primarily dispersed by the air and transmitted by trauma [35,36]. On the contrary, the sporangiospores of *Rhizopus* and *Rhizomucor* species are small (less than 4 µm in diameter), dry and therefore easily airborne [37,38]. This morphological hypothesis does not explain everything because, similar to *Mucor*, *Lichteimia* have wet spores and yet they are equivalently reported in the lung and skin in this review.

#### 3.1.5. Dematiaceous moulds

Dematiaceous fungi are also known as “black fungi” due to the predominance of melanin in their cell walls, which likely acts as a virulence factor. These darkly pigmented fungi are found on the soil surface, where they live as saprophytes but also sometimes as parasites of plants [39]. This review has highlighted 204 dematiaceous fungi species isolated from humans, belonging to 19 genera: *Alternaria*, *Exophiala*, *Cladophialophora*, *Scopulariopsis*, *Curvularia*, *Phialemoniopsis*, *Phialemonium*, *Exserohilum*, *Microascus*, *Bipolaris*, *Chaetomium*, *Cladosporium*, *Ochroconis*, *Phaeoacremonium*, *Rhinocladiella*, *Fonsecaea*, *Phialophora*, *Phoma* and *Madurella*. The genus *Exophiala* is the most represented. Contamination most often takes place through infection of a wound by a telluric strain or during a transcutaneous traumatism by means of a plant [40]. This explains the predominantly cutaneous location found in this review (1043 publications). Among the cutaneous affections, melanised fungi are responsible for chromoblastomycosis, which mainly affect individuals performing soil-related tasks [41], phaeohyphomycosis [42] and eumycotic myetoma [43]. Two synonyms species stand out for their tropism for the central nervous system, *Cladiophialophora bantiana* and *Cladosporium trichoides*, for which 51 publications and 30 publications, respectively, were found in this location.

#### 3.1.6. *Pseudallescheria*/*Scedosporium* Species Complex (PSC)

Among the PSC, six genera were represented: *Allescheria*, *Lomentospora*, *Monosporium*, *Petriellidium*, *Pseudallescheria* and *Scedosporium*. These fungi are ubiquitous in the environment and can be found in soil, compost and polluted water [44]. Regarding current nomenclature and taxonomy, the *Pseudallescheria*/*Scedosporium* species complex includes the three major species isolated from humans: *Scedosporium apiospermum*, *Scedosporium boydii*, and *Lomentospora prolificans*, and four distinct species, namely *Scedosporium aurantiacum* (29 publications), *Scedosporium dehoogii* (2 publications), *Scedosporium inflatum* (86 publications) and *Scedosporium minutisporum* (1 publication) [45]. By grouping the publications concerning the main species and their synonyms, we find 468 publications reporting the isolation of *Scedosporium apiospermum*, 275 publications reporting the isolation of *Scedosporium boydii* and 261 publications concerning the isolation of *Lomentospora prolificans* in humans. In regard to locations, *S. boydii* and *S. apiospermum* are found in all anatomical sites, with a greater prevalence in the pulmonary sphere (72 and 94 publications, respectively) and the cutaneous system (79 and 110 publications, respectively). *Scedosporium prolificans* is mainly found in the pulmonary sphere (56 publications) and in systemic infections (52 publications).

#### 3.1.7. Others

Among the moulds not classified in the five major genera, some present in the environment stands out for their ability to affect multiple organs, such as the members of the genus *Acremonium*, with *Acremonium strictum* (35 publications) and *Acremonium kiliense* (27 publications) in the lead, members of the genus *Paecilomyces*, with *Paecilomyces variotii* (53 publications), and members of the *Trichoderma* genus, with *Trichoderma longibrachiatum* in the lead (40 publications). Others have a cutaneous tropism. *Hendersonula toruloidea* is an opportunistic fungus which is almost exclusively responsible for skin infections (34/35 publications). *Onychocola canadensis* is found only on the skin system (17 publications) and is mostly responsible for onychomycosis (16 publications). Finally, some rarely reported species were exclusively found in the ocular area, often secondary to trauma, such as *Arthrobotrys oligospora* (one publication), *Beauveria alba* (one publication), *Carpoligna pleurothecii* (one publication), *Cephaliophora irregularis* (one publication), *Cephalosporium niveolanosum* (one publication), *Colletotrichum coccodes* (one publication), *Colletotrichum dematium* (seven publications), *Edenia gomezpompae* (one publication), *Epicoccum nigrum* (one publication), *Glomerella cingulate* (one publication), *Laetisaria arvalis* (one publication), *Metarhizium robertsii* (one publication), *Microcyclosporella mali* (one publication), *Paecilomyces viridis* (one publication), *Papulaspora equi* (one publication), *Pestalotiopsis clavispora* (one publication), *Phaeoisaria clematidis* (one publication), *Podospora austroamericana* (one publication), *Pseudopestalotiopsis theae* (one publication), *Roussoella solani* (one publication), *Setosphaeria holmii* (one publication), *Stachybotrys eucylindrospora* (one publication), *Tintelnotia destructans* (one publication) and *Tritirachium roseum* (one publication).

### 3.2. Fungal Location by Focusing on the Anatomical Site

Within the 19 anatomical sites, the semiology of infection was detailed for the six major categories of fungi involved in human pathologies (Table 2).

#### 3.2.1. Systemic

**Figure 2:**
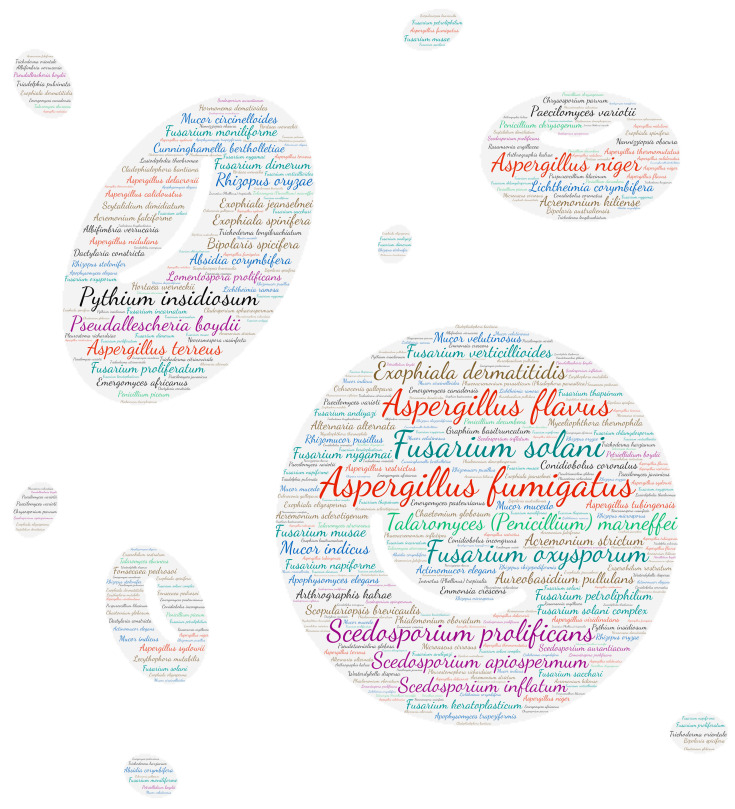
Wordcloud represents the species name involved in systemic infections. The size of the name of each species is proportional to the number of times it occurs in the repertoire.

Regarding systemic localisation (comprising fungaemia, aortitis, vasculitis, lymph node infection and bone marrow infection), there is a large majority of fungaemias. The genus Aspergillus was predominantly isolated from blood samples, with *Aspergillus fumigatus* and *Aspergillus flavus* predominating (Figure 2). It should be noted that the genus *Fusarium* was in second place with *Fusarium solani*. *Aspergillus* is rarely isolated from blood cultures and is usually in the case of infective endocarditis. This predominance of *Aspergillus* detection in systemic infections is explained by the use of new detection tools, including PCR.

#### 3.2.2. Central Nervous System

**Figure 3:**
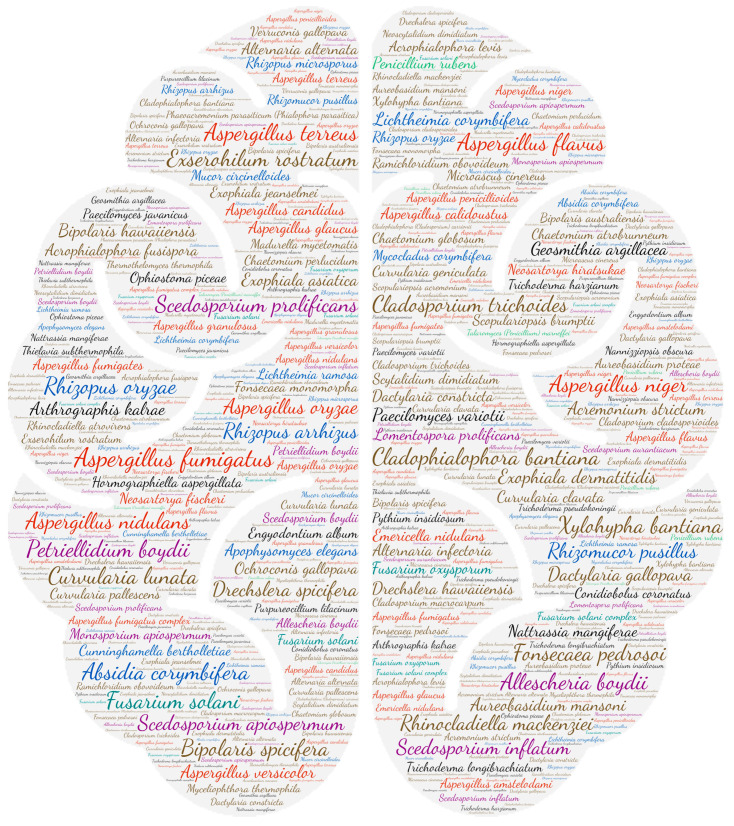
Wordcloud represents the species name isolated in the central nervous system. The size of the name of each species is proportional to the number of times it occurs in the repertoire.

Three genera are mostly represented in central nervous system infections: *Aspergillus*, dematiaceous fungi and *Pseudallescheria*/*Scedosporium* species complex (Figure 3). The majority of infections occur as brain abscesses or meningitis. Figure 2 highlights several species of dematiaceous fungi. *Exserohilum rostratum* has been involved in iatrogenic meningitis outbreaks secondary to the use of contaminated injectable corticosteroids [46,47,48,49,50,51]. *Cladophialophora bantiana* is a dematiaceous mould that may infect immunocompetent patients (mainly farmers or residents of agricultural regions), whose reservoirs and modes of transmission are still poorly known [52]. Its neurotropism is highlighted by 51 publications concerning the CNS among a total of 84 in this review. Its synonym, *Cladosporium trichoides*, causes brain abscesses for which we have recovered 30 publications. Both are responsible for 81 publications reporting CNS involvement.

#### 3.2.3. Ocular System

**Figure 4:**
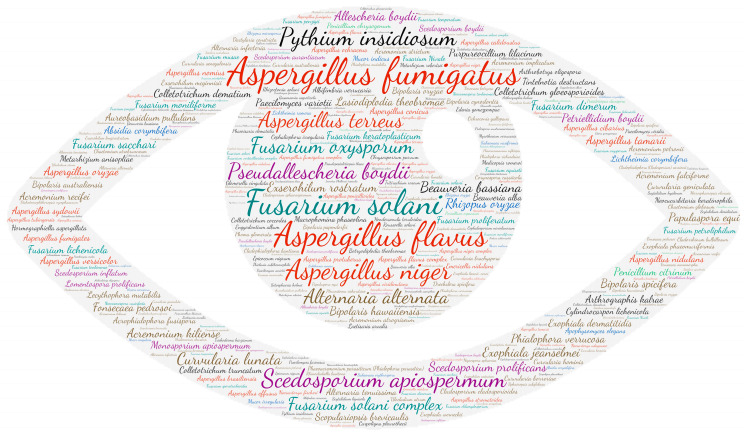
Wordcloud represents the species name isolated in the ocular system. The size of the name of each species is proportional to the number of times it occurs in the repertoire.

All categories of fungi can cause ocular damage. Indeed, as filamentous fungi are ubiquitous in the environment, this type of infection is frequently observed during injuries with plants. The genera *Aspergillus* with *Aspergillus fumigatus*, *Aspergillus flavus*, *Aspergillus terreus* and *Aspergillus niger* and *Fusarium* with *Fusarium solani* and *Fusarium oxysporum* are predominant (Figure 4). In most cases, it can be keratitis (623 publications) or endophthalmitis (240 publications). The most described species that can cause both keratitis and endophthalmitis are *Fusarium solani* (85 and 21 publications, respectively), *Aspergillus fumigatus* (63 and 44 publications, respectively), *Aspergillus flavus* (52 and 16 publications, respectively), *Scedosporium apiospermum* (35 and 13 publications, respectively), *Aspergillus niger* (12 and 13 publications, respectively) and *Pseudallescheria boydii* (10 and 10 publications, respectively). The other species mostly involved in keratitis are *Pythium insidiosum* (31 publications), *Fusarium oxysporum* (21 publications), *Lasiodiplodia theobromae* (11 publications), *Alternaria alternata* (10 publications) and *Colletotrichum gloeosporioides* (10 publications). It should be noted that ten species have been found in conjunctival infections: *Aspergillus flavus* (one), *Aspergillus fumigatus* (one), *Aspergillus niger* (four), *Cephalosporium niveolanosum* (one), *Exophiala jeanselmei* (one), *Fonsecaea pedrosoi* (one), *Monosporium apiospermum* (one), *Neocucurbitaria keratinophila* (one), *Pseudallescheria boydii* (one) and *Scedosporium apiospermum* (one).

#### 3.2.4. Auditory System

**Figure 5:**
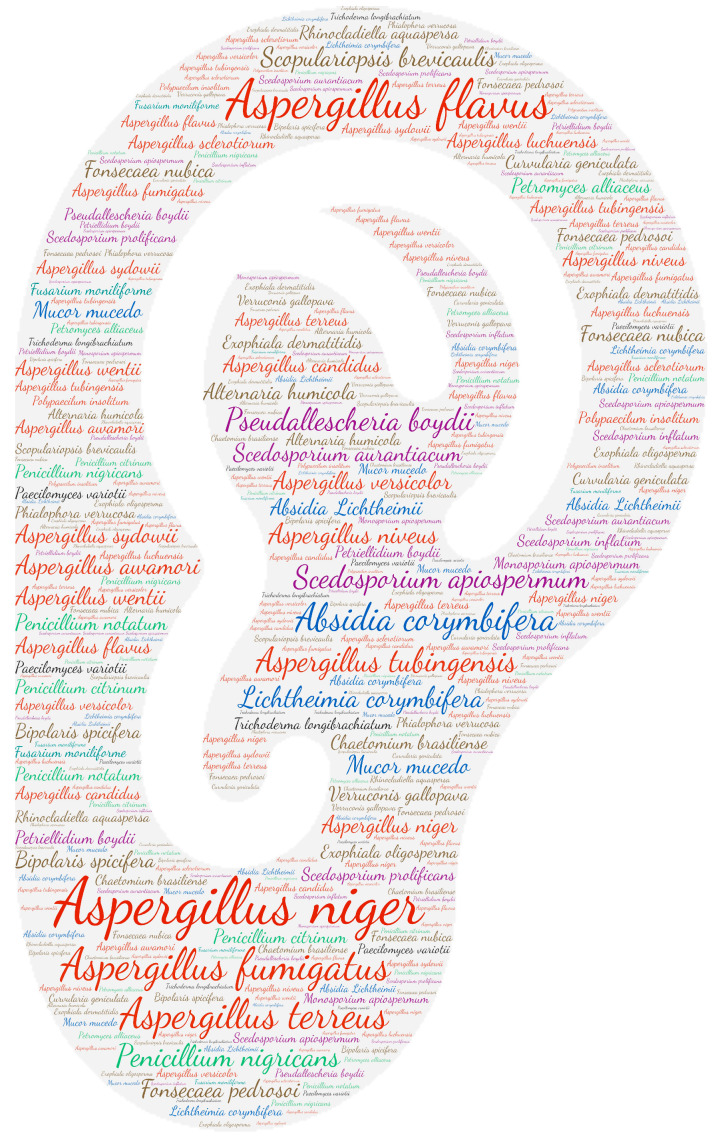
Wordcloud represents the species name isolated in the auditory system. The size of the name of each species is proportional to the number of times it occurs in the repertoire.

The genus *Aspergillus* is predominantly isolated from the auditory system, with *Aspergillus niger* in the lead, a member of the *Nigri* section known for its tropism for the auditory meatus [25]. *Aspergillus flavus* and *Aspergillus fumigatus* come in second place (Figure 5). These fungi are responsible for fungal otomycosis in the majority of cases [53]. Interestingly, 29 publications reported the identification of *Pseudallescheria*/*Scedosporium* complex in the auditory system, including 18 reporting otomycosis. *Scedosporium apiospermum* was predominant, with ten publications reporting otomycosis, including two cases of malignant otitis externa [54,55] and one otitis complicated with temporomandibular arthritis [56]. Other species stand out in Figure 4, such as *Absidia corymbifera*, of which five publications report the involvement in otomycosis, including one case of malignant otitis externa [57] and a few cases of *Penicillium* otomycosis (six publications).

#### 3.2.5. Oto-Rhino-Laryngeal System

**Figure 6:**
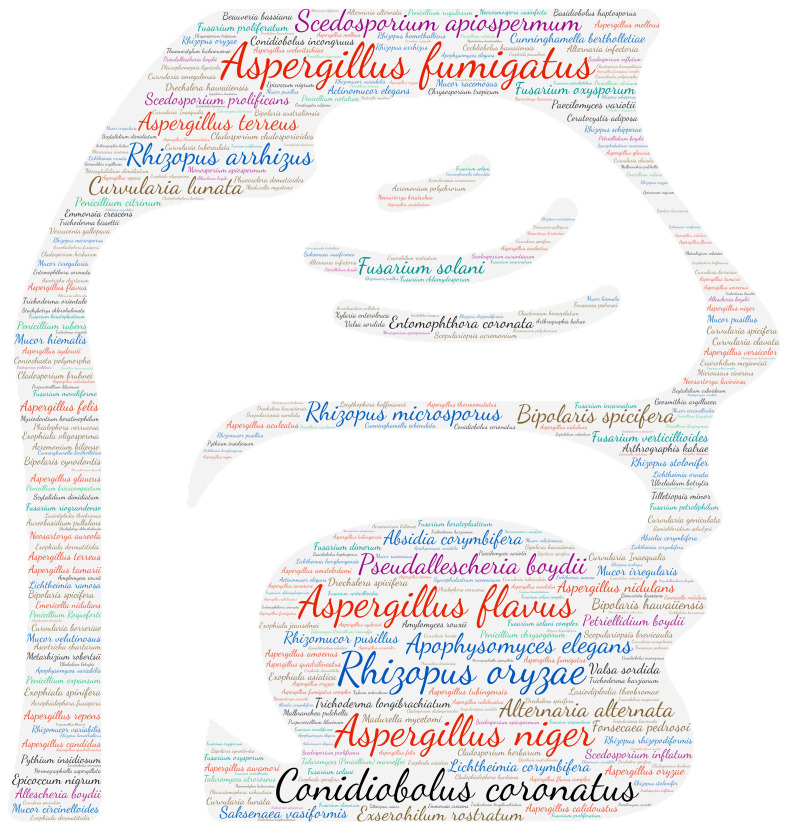
Wordcloud represents the species name isolated in the oto-rhino-laryngeal sphere. The size of the name of each species is proportional to the number of times it occurs in the repertoire.

It is not surprising that a majority of aspergillosis rhinosinusitis is recorded (282 publications) (Figure 6). This anatomical site also includes rhino-orbital, rhino-facial and rhino-orbito-cerebral involvement, which explains the strong representation of mucorales (170 publications). *Rhizopus oryzae* (heterotypic synonym *Rhizopus arrhizus*) is largely in the majority of the mucorales (38 publications), which is not surprising, given that it is the most common mucorale species worldwide [58]. *Rhizopus oryzae* is responsible for variable oto-rhino-laryngeal system infections, with a majority of rhino-orbito-cerebral infections (17 publications) followed by rhino-sinusitis (nine publications) and rhino-orbital involvement (seven publications). These rhino-orbito-cerebral lesions can also be caused by *Aspergillus fumigatus* (16 publications) and two other species of mucorales *Rhizopus arrhizus* (12 publications) and *Apophysomyces elegans* (11 publications). One fungus stands out, *Conidiobolus coronatus*, which is responsible for entomophthoromycosis and rhino-facial infections. *C. coronatus* is a widely distributed insect pathogenic fungus belonging to the class Zygomycetes and is rarely involved in human pathology. This mycosis is mainly tropical due to its tropism of plant detritus in very humid environments [59].

#### 3.2.6. Pulmonary System

**Figure 7:**
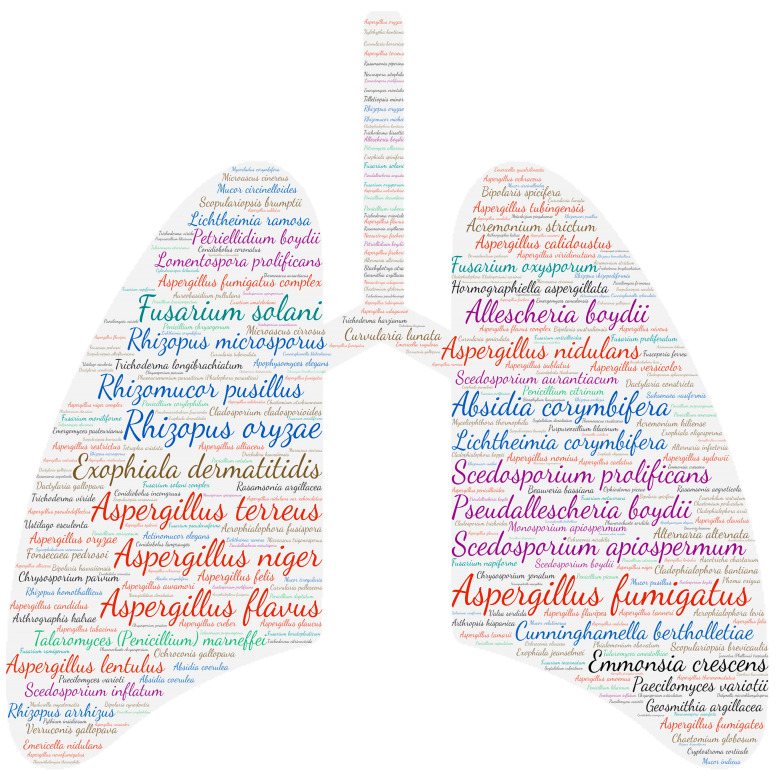
Wordcloud represents the species name isolated in the pulmonary system. The size of the name of each species is proportional to the number of times it occurs in the repertoire.

The respiratory system is the anatomical site most affected by fungal infections (4496 publications). It should be noted that no distinction was made between colonisation and allergic bronchopulmonary aspergillosis, which thus includes the respiratory isolation of *Aspergillus* species. Similar to *Aspergilli*, moulds are ubiquitous saprophytes in the environment. Their dispersion in the air is possible by the production of volatile spores, which ensures their presence in both indoor and outdoor environments [60,61]. Human contamination thus mainly occurs by inhaling the airborne conidia, with the lungs being the first to be exposed. We, therefore, note a wide variety of moulds responsible for lung damage (Figure 7).

#### 3.2.7. Cardiac Involvement

**Figure 8:**
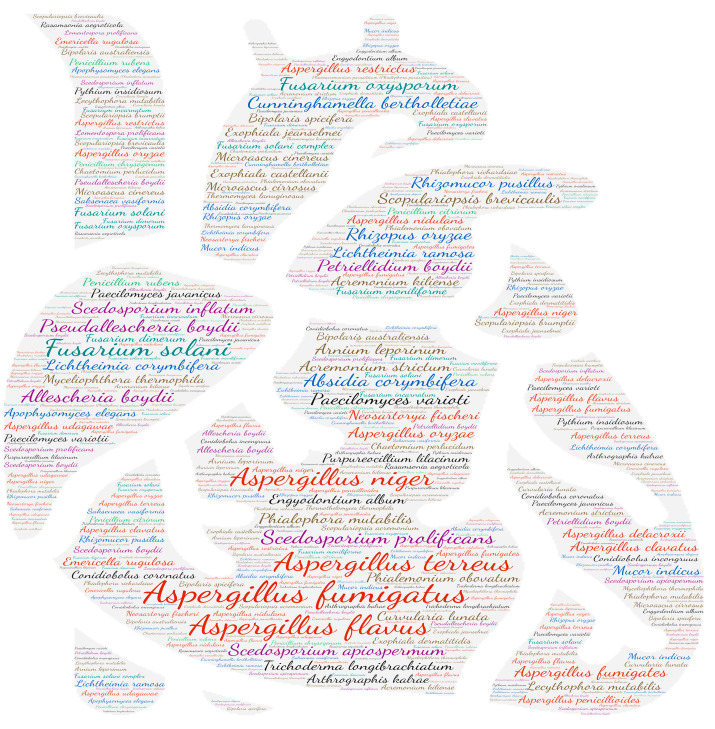
Wordcloud represents the species name isolated in the cardiac system. The size of the name of each species is proportional to the number of times it occurs in the repertoire.

Cardiac involvement was found among 13% (74/565) of the species described in this repertoire. These species belonged to the major taxa *Aspergillus*, *Penicillium*, *Fusarium*, mucorales, dematiaceous fungi and *Scedosporium*/*Lomentospora* complex, as well as the genera *Acremonium* sp, *Arthrographis* sp., *Conidiobolus* sp., *Emericella* sp., *Engyodontium* sp., *Paecilomyces* sp., *Purpureocillium* sp., *Pythium* sp., *Rasamsonia* sp., *Thermothelomyces* sp. and *Trichoderma* sp., with *Aspergillus fumigatus* being the most common species isolated (Figure 8). These infections are, however, rare with species belonging to the genus *Penicillium*, including only three publications (one endocarditis due to *Penicillium chrysogenum* [62] and two pericarditis due to *P. citrinum* and *P. rubens* [63,64]) being reported. Cardiac involvement was mainly in the form of endocarditis, whether native (105 publications) or on a prosthetic valve or implanted equipment (96 publications). Interestingly, in native valve endocarditis, mitral involvement seems to be the most frequent (41 publications) (Table 3). Pericarditis and myocarditis were described in 37 and 49 publications, respectively.

#### 3.2.8. Digestive System

**Figure 9:**
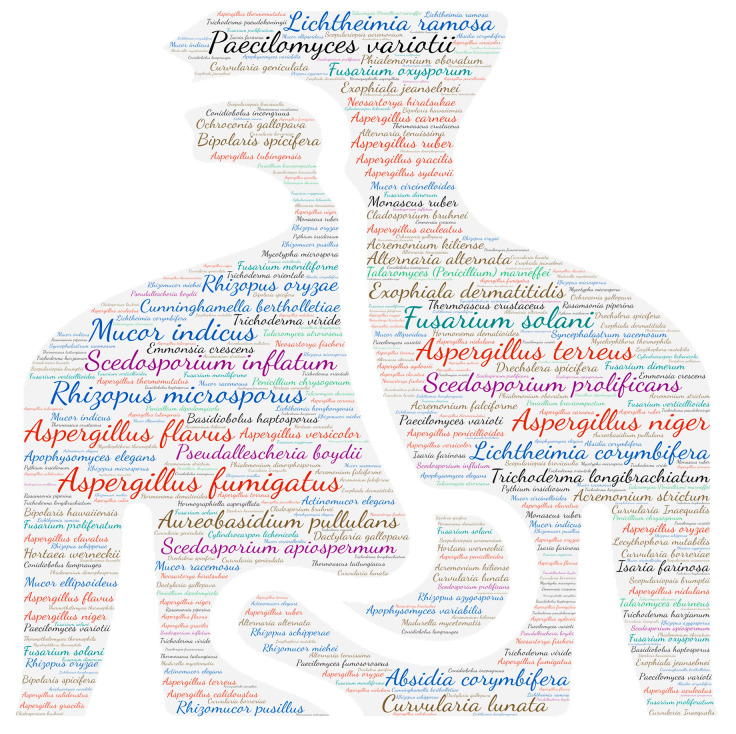
Wordcloud represents the species name isolated in the digestive system. The size of the name of each species is proportional to the number of times it occurs in the repertoire.

Concerning the digestive system, the two main diseases are fungal peritonitis (135 publications) and spleen disease (80 publications) (Figure 9). Micromycetes are known to be responsible for peritonitis during peritoneal dialysis between 1% and 3% of cases, which is indeed the situation we found in the majority of this review [162,163]. In this review, 77.8% of the publications reported peritonitis secondary to peritoneal dialysis (105/135). Other risk factors that emerged were prematurity [164,165,166,167,168] and solid organ transplantation, including the kidney [169,170,171,172,173], heart [174], small bowel [175] and liver [176,177]. Seven publications reported peritoneal involvement secondary to dissemination [174,178,179,180,181,182]. The species mostly found were *Aspergillus fumigatus* (18 publications), *Aspergillus niger* (9 publications) and *Paecilomyces variotii* (8 publications).

Invasive bowel infections by *Aspergillus* and mucorales (14 and 9 publications, respectively) are mainly reported concomitantly with disseminated infection. Invasion of the gastrointestinal tract is rarely described individually [183].

Splenic involvement was, in the majority of cases, secondary to haematogenous dissemination of the pathogen, with the exception of three situations where splenic abscesses were described. One case of a splenic abscess caused by *Aureobasidium pullulans* in a patient with lymphoma [184] and one caused by *Hortaea werneckii* in a patient with acute myeloid leukaemia [185] were both diagnosed postmortem. The third case of splenic abscess caused by *Paecilomyces variotii* was described in a child with chronic granulomatous disease [186].

#### 3.2.9. Liver Involvement

**Figure 10:**
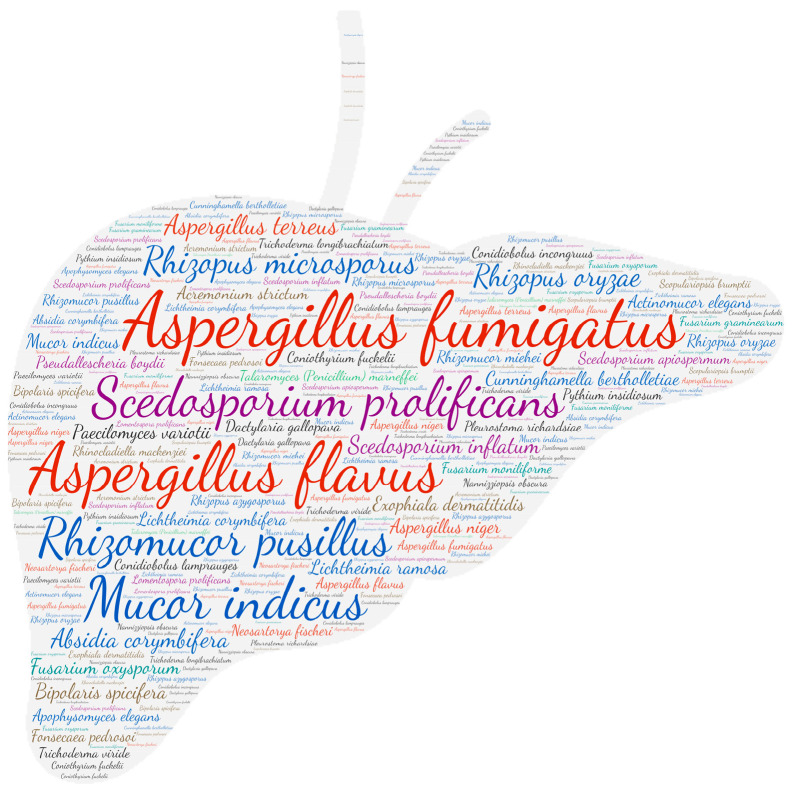
Wordcloud represents the species name isolated in the liver. The size of the name of each species is proportional to the number of times it occurs in the repertoire.

In terms of hepatic involvement (Figure 10), there is great variability in the forms found, including abscesses, hepatitis, ascites and lesions secondary to hematogenous dissemination (Table 2). The genus *Aspergillus* is mostly represented (41 publications) with a predominance of *Aspergillus fumigatus* and *Aspergillus flavus*.

#### 3.2.10. Urinary Tract

**Figure 11:**
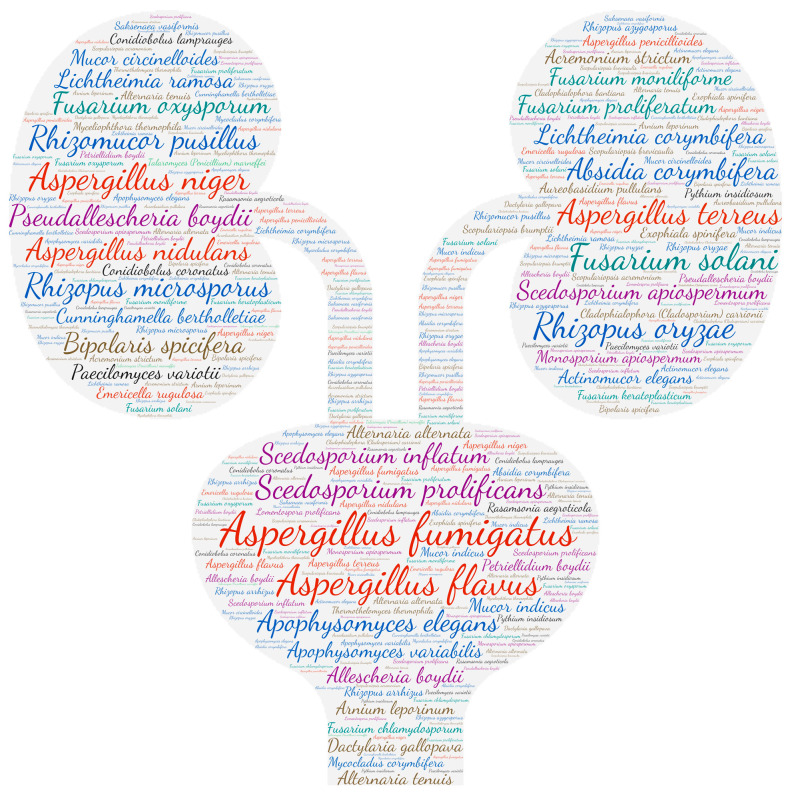
Wordcloud represents the species name isolated in the urinary tract. The size of the name of each species is proportional to the number of times it occurs in the repertoire.

In terms of urinary tract involvement (Figure 11), the vast majority of infections are renal (185 publications), with a predominance of the genus *Aspergillus* (85 publications). These infections may be primary or secondary to the haematogenous dissemination of the fungus.

#### 3.2.11. Osteo-Articular System

**Figure 12:**
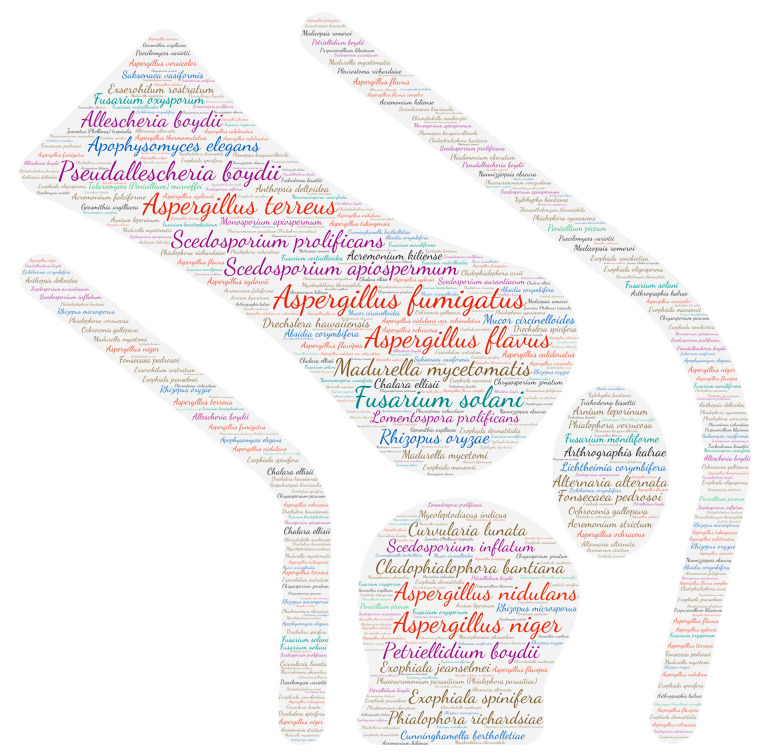
Wordcloud represents the species name isolated in the osteo-articular system. The size of the name of each species is proportional to the number of times it occurs in the repertoire.

Among the osteoarticular diseases, osteomyelitis is the most frequent (266 publications). The second most common form is joint damage (164 publications), which includes arthritis (57 publications), bursitis (one publication), unspecified joint damage (60 publications) and synovitis (46 publications). Spondylodiscitis comes in third place (71 publications). Among the species involved in these infections are species of the genus *Aspergillus* (*Aspergillus fumigatus*, *Aspergillus flavus* and *Aspergillus terreus*), the *Scedosporium*/*Lomentospora* complex (*Pseudallescheria boydii*, *Scedosporium prolificans* and *Scedosporium apiospermum*) and *Fusarium solani* (Figure 12).

#### 3.2.12. Skin System

**Figure 13:**
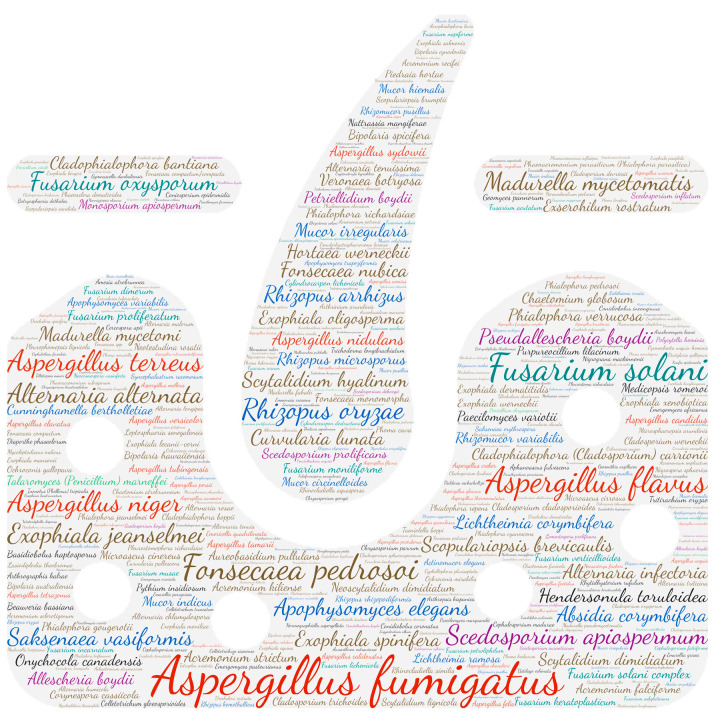
Wordcloud represents the species name isolated in the skin system. The size of the name of each species is proportional to the number of times it occurs in the repertoire.

Concerning the involvement of the cutaneous system, four affections stand out: superficial cutaneous infection (1195 publications), subcutaneous infection (610 publications), onychomycosis (394 publications) and mycetoma (282 publications). *Aspergillus fumigatus* is mostly represented (Figure 13) and is mainly responsible for superficial (84 publications) and subcutaneous (37 publications) infections. The subcutaneous infections are mainly due to dematiaceous fungi (338 publications), with *Exophiala jeanselmei* coming in first position (47 publications). Melanised fungi are responsible for chronic infections, such as chromoblastomycosis, or phaeohyphomycosis, which can evolve towards an invasive character [43]. *Eumycotic mycetoma* are subcutaneous infections that we have chosen to put aside, mainly due to the species *Madurella mycetomatis* (67 publications) and *Madurella mycetomi* (25 publications). Among the agents mainly responsible for onychomycosis, we found *Scopulariopsis brevicaulis* (50 publications), *Hendersonula toruloidea* (28 publications), *Fusarium oxysporum* (22 publications), *Scytalidium dimidiatum* (20 publications), *Aspergillus niger* (18 publications) and *Fusarium solani* (17 publications). These species are responsible for distal and lateral subungual onychomycosis, the most common type of onychomycosis mainly affecting the toenails [187].

#### 3.2.13. Endocrine Glands

Among the endocrine glands, the thyroid is the most frequently affected (66 publications/84). In the majority of cases, the disease is secondary to systemic dissemination of the pathogen, diagnosed postmortem [188]. In fact, infiltration of the thyroid with *Aspergillus* organisms occurs in approximately 20% of autopsies in patients dying from a disseminated disease [189]. However, a few rare cases of primary thyroid infections have been reported with *Aspergillus fumigatus*: two cases of thyroid suppuration in lupus patients treated with corticosteroids [190,191], two cases described in HIV patients [192,193] and one case in a child with chronic granulomatous disease [194]. *Scedosporium apiospermum* was reported to cause multiple thyroid abscesses in a patient with cirrhosis and autoimmune haemolytic anaemia, presenting swelling in the neck [195].

#### 3.2.14. Rarely Involved Anatomical Sites 

*Dental location:* Endodontic infections were rarely found in this review and manifested themselves in the form of root involvement [196,197], granuloma [198,199] or gingival infections [200,201]. No species seemed to have a particular tropism for this sphere.

*Genital sphere*: Interestingly, the male genital sphere seems to be mostly affected, with 71.4% of the publications (15/21) reporting testicular, epididymal or glans involvement. Only three publications reported vaginitis [202,203,204], two reported labial involvement [190,205] and one tubo-ovarian abscess [206]. No species seemed to have a particular tropism for this sphere.

*Breast*: A majority of *Aspergillus* was isolated from this particular site. It should be noted that the *Aspergillus glaucus* and *Aspergillus niger* complexes have already been isolated from milk samples [207,208]. Interestingly, four publications reported fungal infections following breast implant surgery, a situation that is not well-known in this field, due to *Aspergillus niger* [209,210], *Paecilomyces variotii* [211] and *Scedosporium apiospermum* [212].

*Placental infection*: a single case of placental aspergillosis due to *Aspergillus niger* was found in this literature review [213].

## 4. Discussion

The aim of this study was to establish as exhaustive a catalogue as possible of filamentous fungi identified in humans by culture and molecular biology, whether or not they were associated with histopathological findings. We found 565 filamentous fungi identified in humans, for which we specified the organs where these fungi had been found and the semiology of the infections. This repertoire thus helps to understand the pathogenic potential of certain fungi and can also alert clinicians that the isolation of certain rare fungi, such as *Trichoderma longibrachiatum* (from stool specimens), can lead to disseminated infection. One of the limitations of this work, however, is the lack of distinction between colonisation and infection, mainly for fungi isolated from non-sterile sites (i.e., the cutaneous system, pulmonary system, digestive system and ENT sphere). Fungi isolated from sterile sites were considered infections (i.e., the heart, liver and ocular system and CNS). The use of new powerful molecular tools, such as pan-fungal PCR, metagenomic and next-generation sequencing, now means it is possible to detect pathogens even in samples containing extremely low levels of nucleic acids [214,215] and to diagnose mixed infections [216]. However, the application of these tools to medical mycology can lead to interpretation difficulties [217,218,219]. This problem is particularly encountered with filamentous fungi, which are ubiquitous in the environment and for which it is sometimes difficult to distinguish between colonisation, infection and environmental contamination. It is now necessary to go further in our work for each anatomical site in order to distinguish colonisations from infections, in order to help clinicians interpret positive results and to assist with the diagnostic management of patients. Similarly, we did not distinguish between diagnosis by molecular biology and macroscopic identification, although it has been shown that potential errors were found in macroscopic identifications, mostly between close species within a section or species complex [4]. More, even in molecular identification, it was pointed out that 20% of the sequences available in public databases are unreliable [220,221]. We have based our repertoire according to state of the art at the time of reported publications. Therefore, in Table 1, we have chosen to group species by sections, sharing morphological similarities, in order to bypass this limitation. 

Finally, one of the limitations of this publication is its temporality. As explained in Section 2, only references present in PubMed before 16 June 2020 were taken into account in order to have the same PubMed content for all fungi species. However, medical mycology is dynamic, and new organisms constantly need to be accounted for by both clinicians and microbiology laboratories [222]. Moreover, with the COVID-19 pandemic, a new risk factor has emerged [223]. Publications reporting the detection of filamentous fungi in humans have multiplied, reporting, for example, the emergence of mucormycosis and COVID-19-associated pulmonary aspergillosis throughout the world [224,225]. It will therefore be necessary to update this data regularly.

## Figures and Tables

**Table 1:** Number of publications found by anatomical site and species. In the same publication (PMID), several anatomical sites of isolation could be found.

Name	Current Name	Year of 1st Publication	Year of Last Publication	Systemic	CNS	Ocular	Auditory System	Dental and Gums	ORL Sphere	Pulmonary	Breast	Heart	Digestive System	Liver	Urinary Tract	Genital	OA System	Skeletal Muscles	Soft-Tissue	Skin System	Endocrine Gland	Placental Infection	Total
***Aspergillus* spp.**				**181**	**272**	**300**	**192**	**4**	**528**	**1674**	**6**	**198**	**125**	**41**	**122**	**9**	**233**	**21**	**44**	**415**	**35**	**1**	**4401**
**Section *Aspergillus***					**1**	**1**			**1**	**2**	**1**		**1**							**2**			**9**
*Aspergillus chevalieri*		1994	1994																	1			1
*Aspergillus cibarius*		2014	2014			1																	1
*Aspergillus glaucus*		1990	2020		1				1	2	1									1			6
*Aspergillus ruber*		2014	2014										1										1
**Section *Candidi***					**1**		**1**		**1**	**3**										**6**			**12**
*Aspergillus candidus*		1962	2015		1		1		1	3										6			12
**Section *Circumdati***						**1**	**1**		**1**	**2**							**1**		**1**	**4**			**11**
*Aspergillus melleus*		2015	2020						1											1			2
*Aspergillus ochraceopetaliformis*		2009	2009																	1			1
*Aspergillus ochraceus*		1987	2019			1				2							1		1				5
*Aspergillus persii*		2010	2010																	1			1
*Aspergillus sclerotiorum*		2004	2004				1																1
*Aspergillus subramanianii*		2018	2018																	1			1
**Section *Clavati***										**1**		**1**	**1**							**2**			**5**
*Aspergillus clavatus*		1986	2019							1		1	1							2			5
**Section *Cremei***						**1**	**1**																**2**
*Aspergillus stromatoides*		1987	1987			1																	1
*Aspergillus wentii*		2011	2011				1																1
**Section *Flavi***				**32**	**43**	**93**	**47**		**166**	**190**	**1**	**45**	**22**	**7**	**33**	**1**	**58**	**5**	**14**	**104**	**4**		**865**
*Aspergillus alliaceus*		2007	2010							2													2
*Aspergillus caelatus*		2019	2019							1													1
*Aspergillus effusus*		2019	2019			1																	1
*Aspergillus flavus*		1956	2020	32	41	81	47		162	174	1	44	21	7	33	1	58	5	14	98	4		823
*Aspergillus flavus complex*		2006	2020			3				3													6
*Aspergillus minisclerotigenes*		2014	2014						1														1
*Aspergillus nomius*		2009	2020			1				3										1			5
*Aspergillus oryzae*		1976	2018		2	3			2	5		1	1										14
*Aspergillus tamarii*		1992	2020			4			1	1										5			11
*Aspergillus tanneri*		2012	2012							1													1
**Section *Flavipedes***										**2**							**1**						**3**
*Aspergillus flavipes*		1979	1999							2							1						3
**Section *Fumigati***				**116**	**194**	**140**	**46**	**1**	**293**	**1225**		**118**	**71**	**30**	**73**	**7**	**133**	**14**	**21**	**161**	**28**		**2671**
*Aspergillus felis*		2013	2019				0		1	4										1			6
*Aspergillus fischeri*		1973	1998							2													2
*Aspergillus fumigatus*		1945	2022	114	190 (191)	134 (136)	46	1	287	1180 (1186)		113 (115)	67	29	73	7	131	14	21	156	27		2590 (2601)
*Aspergillus fumigates*	*Aspergillus fumigatus*	2010	2020		1	2				6		2											11
*Aspergillus fumigatus complex*		2012	2020	0	1	1			1	10							1			1			15
*Aspergillus lentulus*		2006	2020							14													14
*Aspergillus novofumigatus*		2013	2013							1													1
*Aspergillus thermomutatus*		1994	2020	1					1	1			1				1			2			7
*Aspergillus udagawae*		2012	2017							1		1											2
*Aspergillus viridinutans*		2009	2014	1		1				4													6
*Neosartorya aureola*	*Aspergillus aureoluteus (anamorph)*	2011	2017						1														1
*Neosartorya fischeri*	*Aspergillus fischeranus (anamorph)*	1990	1997		1	1				1		2	1	1						1	1		9
*Neosartorya hiratsukae*	*Aspergillus hiratsukae (anamorph)*	2002	2010		1				1				2										4
*Neosartorya laciniosa*	*Aspergillus laciniosus (anamorph)*	2013	2013						1														1
*Neosartorya udagawae*	*Aspergillus udagawae (anamorph)*	2009	2011			1				1													2
**Section *Nidulantes***				**3**	**12**	**10**	**5**	**1**	**12**	**39**	**1**	**3**	**4**		**4**	**1**	**14**	**2**	**4**	**40**			**155**
*Aspergillus amoenus*		2016	2016						1	1													2
*Aspergillus creber*		2016	2016							1										1			2
*Aspergillus delacroxii*		2015	2015	1								1											2
*Aspergillus hongkongensis*		2016	2016																	1			1
*Aspergillus nidulans*		1963	2021	1	9 (10)	3 (4)	2		6 (8)	20 (23)	1	1	1		3		11	2	4	19			83 (90)
*Emericella nidulans*	*Aspergillus nidulans*	1983	2016		1	1			2	3													7
*Aspergillus nidulans var. echinulatus*	*Aspergillus delacroxii*	1988	1988							1							1						2
*Aspergillus protuberus*		2015	2019			1										1				1			3
*Aspergillus quadrilineatus*		1992	1992						1	(1)										(4)			1 (6)
*Aspergillus tetrazonus*	*Aspergillus quadrilineatus*	2004	2015																	2			2
*Emericella quadrilineata*	*Aspergillus quadrilineatus*	2004	2015							1										2			3
*Aspergillus sydowii*		1989	2019	1		2	1		1	2			1				1			7			16
*Aspergillus sublatus*		2014	2019							3													3
*Aspergillus tabacinus*		2016	2016							1													1
*Aspergillus unguis*		2016	2016																	1			1
*Aspergillus versicolor*		1950	2021		2	3	2	1	1	5			2				1			5			22
*Emericella rugulosa*	*Aspergillus rugulovalvus*	2012	2012							1		1			1					1			4
**Section *Nigri***				**17**	**4**	**36**	**79**	**1**	**33**	**113**	**3**	**12**	**12**	**1**	**4**		**9**		**4**	**58**		**1**	**387**
*Aspergillus aculeatus*		1984	1984						1				1										2
*Aspergillus awamori*		1992	2020				1		1	2										1			5
*Aspergillus brasiliensis*		2010	2020			1														1			2
*Aspergillus luchuensis*		2010	2010				1																1
*Aspergillus niger*		1965	2020	14	4	33	72	1	28	102	2	12	10	1	4		8		4	47 (48)		1	343 (344)
*Aspergillus foetidus*	*Aspergillus niger*	1992	1992																	1			1
*Aspergillus niger complex*		2002	2014			1				1	1												3
*Aspergillus tubingensis*		2009	2020	3		1	5		2	7			1				1			6			26
*Aspergillus uvarum*		2015	2015																	1			1
*Aspergillus welwitschiae*		2016	2019						1	1										1			3
**Section *Polypaecilum***							**1**																**1**
*Polypaecilum insolitum*	*Aspergillus insolitus*	1972	1972				1																1
**Section *Restricti***				**1**	**1**	**2**				**4**		**3**	**2**		**1**								**14**
*Aspergillus conicus*		2013	2013			1																	1
*Aspergillus gracilis*		2019	2019										1										1
*Aspergillus penicillioides*		2016	2018		1	1				1		1	1		1								6
*Aspergillus restrictus*		1960	1993	1						3		2											6
**Section *Terrei***				**11**	**13**	**15**	**11**	**1**	**16**	**82**		**16**	**11**	**3**	**7**		**16**			**35**	**3**		**240**
*Aspergillus carneus*		2016	2016										1										1
*Aspergillus niveus*		1984	2008				1			1													2
*Aspergillus terreus*		1948	2021	11	13	15	10	1	16	81		16	10	3	7		16			35	3		237
**Section *Usti***				**1**	**2**	**1**			**3**	**10**			**1**				**1**			**3**			**22**
*Aspergillus calidoustus*		2008	2021	1	1	1			3	9			1				1			2			19
*Aspergillus granulosus*		1995	2009		1															1			2
*Aspergillus pseudodeflectus*		2018	2018							1													1
**Unknown Section**					**1**				**2**	**1**													**4**
*Aspergillus amstelodami*		1983	2008		1				1														2
*Aspergillus repens*		1989	1989						1														1
*Eurotium amstelodami*		2016	2016							1													1
***Penicillium* spp.**				**36**	**3**	**8**	**5**	**1**	**17**	**48**	**1**	**3**	**11**	**2**	**1**		**4**			**23**			**163**
**Section *Biverticillata***				**1**						**1**							**1**						**3**
*Penicillium piceum*		2001	2006	1						1							1						3
**Section *Brevicompacta***									**1**	**1**			**1**										**3**
*Penicillium brevicompactum*		1996	2013						1	1			1										3
**Section *Canescentia***							**3**																**3**
*Penicillium nigricans*		1992	2002				3																3
**Section *Chrysogena***				**2**	**2**	**2**	**1**		**5**	**5**	**1**	**2**	**2**							**2**			**24**
*Penicillium chrysogenum*		1974	2016	2	1	2	(1)		3 (4)	3 (4)		1	2							2			16 (19)
*Penicillium notatum*	*Penicillium chrysogenum*	1978	2016				1		1	1													3
*Penicillium rubens*		2016	2020		1				1	1	1	1											5
**Section *Citrina***						**5**	**1**	**1**	**3**	**4**		**1**											**15**
*Penicillium citrinum*		1976	2020			4 (5)	1	(1)	3	4		1											13 (15)
*Penicillium implicatum*	*Penicillium citrinum*	2010	2010			1		1															2
**Section *Exilicaulis***				**1**						**3**													**4**
*Penicillium corylophilum*		2002	2011							2													2
*Penicillium decumbens*		1990	1992	1						1													2
**Section *Fasciculata***										**1**													**1**
*Penicillium verrucosum*		2001	2001							1													1
**Section *Lanata-Divaricata***																				**1**			**1**
*Penicillium vitale*	*Penicillium simplicissimum*	1992	1992																	1			1
**Section *Penicillium***						**1**			**1**	**1**													**3**
*Penicillium digitatum*		2013	2013							1													1
*Penicillium expansum*		1978	2000			1			1														2
**Section *Ramigena***										**1**													**1**
*Penicillium capsulatum*		2013	2013							1													1
**Section *Robsamsonia***													**1**										**1**
*Penicillium dipodomyicola*		2013	2013										1										1
**Section *Roquefortorum***									**1**														**1**
*Penicillium roqueforti*		2018	2018						1														1
**Section *Talaromyces***				**30**	**1**				**4**	**25**			**5**	**2**	**1**		**3**			**19**			**90**
*Talaromyces (Penicillium) marneffei*		2015	2020	30	1				4	24			5	2	1		3			19			89
*Talaromyces purpurogenus*		2016	2016							1													1
**Section *Tachyspermi***				**1**					**1**	**3**			**1**							**1**			**7**
*Talaromyces amestolkiae*		2016	2017							2													2
*Talaromyces atroroseus*		2020	2020	1					1	1			1							1			5
**Unclassified into a Section**				**1**					**1**	**3**			**1**										**6**
*Penicillium lilacinum*		1972	1972							1													1
*Penicillium purpurogenum*		1998	1998							1													1
*Penicillium rugulosum*	*Talaromyces rugulosus*	1999	1999						1														1
*Talaromyces eburneus*		2010	2010	1						1			1										3
***Fusarium* spp.**				**134**	**2**	**190**	**1**	**1**	**34**	**41**	**1**	**12**	**17**	**4**	**14**	**1**	**16**	**1**	**4**	**233**	**1**		**713**
***F. chlamydosporum Species Complex (FCSC)***				**1**		**1**			**1**						**1**					**1**			**5**
*Fusarium chlamydosporum*		1985	2020	1		1			1						1					1			5
***F. dimerum Species Complex (FDSC)***				**2**		**7**			**1**			**1**	**1**						**1**	**4**			**17**
*Fusarium dimerum*		1972	2018	2		6			1			1	1						1	4			16
*Fusarium penzigii*		2016	2016			1																	1
***F. incarnatum–F. equiseti Species Complex (FIESC)***				**1**		**5**			**1**	**1**		**1**								**3**			**12**
*Fusarium equiseti*		2007	2007			1																	1
*Fusarium incarnatum*		2014	2020	1		4			1	1		1								3			11
***F. oxysporum Species Complex (FOSC)***				**21**	**2**	**24**			**5**	**10**		**3**	**4**	**3**	**3**		**3**			**53**			**131**
*Fusarium oxysporum*		1958	2021	21	2	24			5	10		3	4	3	3		3			53			131
***F. sambucinum Species Complex (FSAMSC)***						**1**														**2**			**3**
*Fusarium roseum*	*Fusarium sambucinum*	1987	1987																	1			1
*Fusarium sporotrichioides*		2017	2017			1														1			2
***F. solani Species Complex (FSSC)***				**81**		**138**			**20**	**24**	**1**	**6**	**9**		**8**	**1**	**10**	**1**	**3**	**143**	**1**		**452**
*Phialophora cyanescens*	*Neocosmospora cyanescens*	1984	1993														1			3			4
*Cephalosporium falciforme*	*Neocosmospora (fusarium) falciformis*	1968	1983																	2			2
*Fusarium keratoplasticum*		2015	2019	7		8			2	1					1		1			9			29
*Fusarium lichenicola*		2003	2020			3														2			5
*Fusarium metavorans*		2018	2018							1													1
*Fusarium petroliphilum*		2013	2019	6		2			1											3			12
*Fusarium proliferatum*		1988	2020	6		3			3	2			1		3				1	14			33
*Fusarium pseudensiforme*	*Neocosmospora pseudensiformis*	2020	2020							1													1
*Fusarium riograndense*		2018	2018						1														1
*Fusarium solani*		1970	2020	55	5	102			10	15	1	5	8		4	1	7	1	2	94	1		311
*Fusarium solani complex*		2005	2019	6	1	18			2	3		1								14			45
*Neocosmospora tonkinensis*		2018	2018			1																	1
*Neocosmospora vasinfecta*	*Fusarium neocosmosporiellum*	1993	2008	1		1			1	1							1			2			7
***F. fujikuroi Species Complex (FFSC)***				**2**		**1**														**2**			**5**
*Fusarium musae*		2015	2016	2		1														2			5
***Gibberella fujikuroi Species Complex (GFSC)***				**26**		**12**	**1**	**1**	**6**	**6**		**1**	**3**	**1**	**2**		**3**			**25**			**87**
*Fusarium acutatum*		2006	2015																	2			2
*Fusarium andiyazi*		2014	2014	1																			1
*Fusarium moniliforme*		1977	2013	7		6	1	1	1	2		1	2	1	2		2			11			37
*Fusarium napiforme*		1993	2018	2						2										2			6
*Fusarium nygamai*		1996	2015	3																			3
*Fusarium ramigenum*		2016	2016							1													1
*Fusarium sacchari*		2000	2020	1		4														1			6
*Fusarium subglutinans*		2010	2013																	2			2
*Fusarium temperatum*		2014	2014			1																	1
*Fusarium thapsinum*		2004	2004	1																			1
*Fusarium verticillioides*		1995	2018	11					5	1			1				1			7			26
*Fusarium verticillioides complex*		2013	2013			1																	1
**Unclassified Into Complex**						**1**																	**1**
*Fusarium langsethiae*		2015	2015			1																	1
**Mucorales**				**45**	**40**	**18**	**13**	**0**	**164**	**189**	**1**	**26**	**69**	**34**	**50**	**6**	**25**	**24**	**50**	**321**	**14**		**1089**
***Absidia***							**1**			**2**													**3**
*Absidia coerulea*		1988	1988							2													2
*Absidia Lichtheimii*		1968	1968				1																1
***Actinomucor***				**1**					**2**	**2**			**1**	**1**	**1**			**1**		**3**	**1**		**13**
*Actinomucor elegans*		2001	2020	1					2	2			1	1	1			1		3	1		13
***Apophysomyces***				**3**	**1**	**1**			**23**	**3**		**1**	**5**	**1**	**14**		**7**	**9**	**19**	**54**			**141**
*Apophysomyces elegans*		1985	2020	2	1	1			19	3		1	3	1	12		7	7	12	41			110
*Apophysomyces trapeziformis*		2012	2014	1														2	5	4			12
*Apophysomyces variabilis*		2011	2020						4				2		2				2	9			19
***Cunninghamella***				**10**	**4**				**6**	**46**		**9**	**5**	**3**	**3**		**3**	**1**	**3**	**12**	**4**		**109**
*Cunninghamella bertholletiae*		1979	2019	10	4				5	44		9	5	3	3		3	1	3	11	4		105
*Cunninghamella blakesleeana*		2012	2012							1													1
*Cunninghamella echinulata*		2009	2013						1	1										1			3
***Lichtheimia***				**14**	**19**	**9**	**9**		**23**	**51**		**9**	**20**	**8**	**13**	**2**	**4**	**3**	**9**	**77**	**6**		**276**
*Lichtheimia corymbifera*		1982	2019	9 (13)	9 (18)	4 (8)	4 (9)		9 (20)	22 (43)		3 (7)	9 (14)	3 (6)	6 (11)	1 (2)	3 (4)	1 (3)	3 (9)	37 (67)	2 (4)		125 (238)
*Absidia corymbifera*	*Lichtheimia corymbifera*	1982	2015	4	8	4	5		11	20		4	5	3	4	1	1	2	6	29	2		109
*Mycocladus corymbifera*	*Lichtheimia corymbifera*	2013	2013		1					1					1					1			4
*Lichtheimia hongkongensis*		2010	2010						1				1							1			3
*Lichtheimia ornata*		2018	2020						1											1			2
*Lichtheimia ramosa*		2010	2019	1	1	1			1	8		2	5	2	2					8	2		33
***Mucor***				**9**	**1**	**2**	**1**		**11**	**7**		**1**	**10**	**5**	**3**	**1**	**2**	**2**		**40**			**95**
*Mucor circinelloides*		1987	2019	3	1				2	4			1		2	1	2			7			23
*Mucor ellipsoideus*	*Mucor ardhlaengiktus*	2011	2011										1										1
*Mucor hiemalis*		1986	2015						1											4			5
*Mucor indicus*		1990	2019	2		1				1		1	7	5	1			2		5			25
*Mucor irregularis*		2011	2019			1			3 (6)	1										12 (21)			17 (29)
*Rhizomucor variabilis*	*Mucor irregularis*	2009	2018						3											9			12
*Mucor lusitanicus*		1990	1990																	1			1
*Mucor mucedo*		1966	2013	1			1																2
*Mucor racemosus*		2011	2015						1				1										2
*Mucor ramosissimus*		1993	1993																	1			1
*Mucor velutinosus*		2011	2018	3					1	1										1			6
***Rhizomucor***				**2**	**5**				**10**	**21**		**3**	**4**	**7**	**5**	**1**	**1**			**8**	**2**		**69**
*Rhizomucor miehei*		1999	2017							2		0	1	1						1			5
*Rhizomucor pusillus*		1983	2017	2	5				9 (10)	18 (19)		3	3	6	5	1	1			6 (7)	2		61 (64)
*Mucor pusillus*	*Rhizomucor pusillus*	1952	1981						1	1										1			3
***Rhizopus***				**6**	**10**	**4**			**80**	**54**	**1**	**2**	**21**	**9**	**10**	**2**	**6**	**4**	**13**	**75**			**297**
*Rhizopus arrhizus*		1975	2020	(3)	4 (9)	(3)			19 (57)	8 (29)	(1)	(2)	(4)	(4)	1 (4)	(1)	(4)	2 (3)	3 (7)	16 (44)			53 (175)
*Rhizopus oryzae*	*Rhizopus arrhizus*	1955	2020	3	5	3			38	21	1	2	4	4	3	1	4	1	4	27			121
*Rhizopus delemar*	*Rhizopus arrhizus var. delemar*	2014	2014																	1			1
*Rhizopus homothallicus*		2010	2019						2	4									0	2			8
*Rhizopus microsporus*		1988	2020	1	1	1			15	19			15 (16)	4 (5)	5 (6)	1	2	1	5	20 (22)			90 (95)
*Rhizopus azygosporus*	*Rhizopus microsporus*	1996	2005										1	1	1					1			4
*Rhizopus oligosporus*	*Rhizopus microsporus var. oligosporus*	1989	1989																	1			1
*Rhizopus rhizopodiformis*	*Rhizopus microsporus var. rhizopodiformis*	1978	2008	1					3	2									1	6			13
*Rhizopus pusillus*		2019	2019																	1			1
*Rhizopus schipperae*		1999	1999						1				1										2
*Rhizopus stolonifer*		1996	2018	1					2														3
***Saksenaea***						**2**	**2**		**6**	**2**		**1**			**1**		**2**	**4**	**6**	**45**	**1**		**72**
*Saksenaea erythrospora*		2011	2018			1													3	4			8
*Saksenaea vasiformis*		1976	2020			1	2		6	2		1			1		2	4	3	41	1		64
***Syncephalastrum***									**2**	**1**			**3**							**6**			**12**
*Syncephalastrum racemosum*		2005	2020						2	1			3							6			12
***Thamnostylum***									**1**														**1**
*Thamnostylum lucknowense*		2012	2012						1														1
***Thermomucor***																				**1**			**1**
*Thermomucor indicae-seudaticae*		1993	1993																	1			1
**Dematiaceous Fungi**				**69**	**189**	**160**	**17**	**5**	**127**	**166**	**3**	**28**	**45**	**10**	**14**	**1**	**70**	**8**	**15**	**1043**	**6**		**1976**
***Alternaria***				**1**	**2**	**16**	**1**		**15**	**5**			**4**		**2**		**2**		**2**	**84**			**134**
*Alternaria alternata*		1976	2019	1	1	13			13	4			3		1 (2)		2		2	40 (41)			80 (82)
*Alternaria tenuis*	*Alternaria alternata*	1960	1970												1					1			2
*Ulocladium atrum*	*Alternaria atra*	2006	2006			1																	1
*Alternaria chlamydospora*		1990	2001																	4			4
*Alternaria dennisii*		2016	2016																	1			1
*Alternaria humicola*		1984	1985				1													1			2
*Alternaria infectoria*		1998	2020		1	1			2	1										25			30
*Alternaria longipes*		1995	1995																	1			1
*Alternaria malorum*		2012	2013																	1			1
*Alternaria rosae*		2017	2017																	1			1
*Alternaria tenuissima*		1986	2020			1							1							8			10
*Alternaria triticina*		2014	2014																	1			1
***Exophiala***				**25**	**10**	**19**	**2**	**2**	**9**	**44**	**1**	**3**	**9**	**3**	**1**	**1**	**11**	**1**	**3**	**172**	**1**		**317**
*Exophiala asiatica*		2009	2009		1				1														2
*Exophiala bergeri*		2016	2016																	1			1
*Exophiala castellanii*		1994	1994									1											1
*Exophiala dermatitidis*		1984	2020	17	5	6	1		5	38	1	1	6	3			1	1	2	9	1		97
*Exophiala equina*		2013	2013																	1			1
*Exophiala hongkongensis*		2013	2013																	1			1
*Exophiala jeanselmei*		1981	2020	3	1 (4)	10		2	1	3		1	3			1	3 (4)			85 (98)			113 (130)
*Exophiala mansonii*	*Exophiala jeanselmei var. castellanii*	1986	1989														1			2			3
*Aureobasidium mansoni*	*Exophiala jeanselmei var. castellanii*	1989	1998		3															1			4
*Phialophora gougerotii*	*Exophiala jeanselmei*	1967	1983																	6			6
*Phialophora jeanselmei*	*Exophiala jeanselmei*	1964	1979																	4			4
*Exophiala lecanii-corni*		1994	2018																	5			5
*Exophiala moniliae*		1981	1984																	2			2
*Exophiala oligosperma*		2003	2020	1			1		1	2							1		1	16			23
*Exophiala phaeomuriformis*		2017	2018			3																	3
*Exophiala pisciphila*		1991	1991																	1			1
*Exophiala polymorpha*		2015	2015																	1			1
*Exophiala salmonis*		2006	2012																	2			2
*Exophiala spinifera*		1983	2020	4					1	1					1		4			29			40
*Exophiala xenobiotica*		2009	2016														1			6			7
***Cladophialophora***				**1**	**92**	**2**			**1**	**10**					**1**		**7**		**1**	**75**			**190**
*Cladophialophora ajelloi*	*Cladophialophora carrionii*	1982	1982																	1			1
*Cladophialophora arxii*		2009	2015							1							1						2
*Cladophialophora bantiana*		1996	2020	1	51 (92)	1			1	5 (7)							5 (6)		1	19 (25)			84 (134)
*Cladosporium trichoides*	*Cladophialophora bantiana*	1952	2020		30					1										6			37
*Xylohypha bantiana*	*Cladophialophora bantiana*	1989	2013		11					1							1						13
*Cladophialophora boppii*		2009	2020							1										5 (7)			6 (8)
*Taeniolella boppi*	*Cladophialophora boppii*	1983	1983																	2			2
*Cladophialophora (Cladosporium) carrionii*		1979	2021			1				1					1					38			41
*Cladophialophora devriesii*		2006	2006																	1 (3)			1 (3)
*Cladosporium devriesii*	*Cladophialophora devriesii*	1984	1990																	2			2
*Cladophialophora saturnica*		2009	2009																	1			1
***Scopulariopsis***				**6**	**3**	**7**	**4**		**6**	**16**		**7**	**3**	**1**	**3**		**1**		**1**	**80**	**2**		**140**
*Scopulariopsis acremonium*		1998	2009		1				2	1		1	1		1						1		8
*Scopulariopsis alboflavescens*		2018	2018							1										1			2
*Scopulariopsis brevicaulis*		1951	2020	6		7	4		3	7		5	1		1		1		1	72			108
*Scopulariopsis brumptii*		1975	2017		2					6		1	1	1	1					5	1		18
*Scopulariopsis candida*		1994	2015						1	1										2			4
***Curvularia***					**10**	**20**	**1**		**26**	**11**		**1**	**10**				**6**		**2**	**29**			**116**
*Curvularia australiensis*		2015	2015			1																	1
*Curvularia borreriae*		2013	2013			1			1	1			1										4
*Curvularia brachyspora*		1992	1997			1														1			2
*Curvularia clavata*		1999	2009		1				1											1			3
*Curvularia geniculata*		1964	2014		1	3	1		1	1			2							1			10
*Drechslera hawaiiensis*	*Curvularia hawaiiensis*	1973	1999		2				2	1							2						7
*Curvularia hominis*		2018	2018			1																	1
*Curvularia inaequalis*		2005	2013						2				1							1			4
*Curvularia lunata*		1970	2019		3	11			13	6		1	4				3		2	20			63
*Curvularia pallescens*		1977	1995		1					1										2			4
*Curvularia senegalensis*		1991	1999			1			1											1			3
*Curvularia spicifera*		2017	2017		(2)	(1)			1 (4)				(2)				(1)			(1)			1 (11)
*Drechslera spicifera*	*Curvularia spicifera*	1975	1988		2	1			3				2				1			1			10
*Curvularia tuberculata*		2019	2019						1	1										1			3
***Phialemoniopsis***																				**2**			**2**
*Phialemoniopsis endophytica*		2017	2017																	1			1
*Phialemoniopsis hongkongensis*		2014	2014																	1			1
***Phialemonium***				**3**		**1**				**2**		**2**	**2**				**1**			**3**			**14**
*Phialemonium dimorphosporum*	*Phialemonium curvatum*	1993	1999	1																1			2
*Phialemonium obovatum*		1986	2012	2		1				2		2	2				1			2			12
***Exserohilum***				**1**	**10**	**10**			**11**	**1**							**2**			**14**			**49**
*Exserohilum longirostratum*		1994	2006			1														1			2
*Exserohilum mcginnisii*		1986	2018			2			1														3
*Exserohilum rostratum*		1986	2020	1	10	7			10	1							2			13			44
***Microascus***				**1**	**1**				**1**	**8**		**2**								**8**			**21**
*Microascus cinereus*		1980	2013		1				1	2		1								4			9
*Microascus cirrosus*		1992	2018	1						4		1								3			9
*Microascus ennothomasiorum*		2019	2019																	1			1
*Microascus trigonosporus*		2004	2015							2													2
***Bipolaris***				**5**	**8**	**18**	**1**		**22**	**9**	**1**	**2**	**3**	**2**	**2**			**1**		**15**	**2**		**91**
*Bipolaris australiensis*		1986	2017	1	1	3			1	1		1								2			10
*Bipolaris cynodontis*		2012	2015			1			1	1										2			5
*Bipolaris hawaiiensis*		1986	2019		2	8			5	2			1							4			22
*Bipolaris oryzae*		2016	2016			1																	1
*Bipolaris papendorfii*		2005	2005			1																	1
*Bipolaris spicifera*		1984	2015	4	5	4	1		15	5	1	1	2	2	2			1		7	2		52
***Chaetomium***				**1**	**4**	**2**	**1**		**1**	**6**		**1**								**16**			**32**
*Chaetomium atrobrunneum*		1998	2019		2	1				1										3			7
*Chaetomium brasiliense*		2011	2011				1																1
*Chaetomium funicola*		2007	2007																	1			1
*Chaetomium globosum*		1988	2020	1	1	1				4										12			19
*Chaetomium homopilatum*	*Humicola homopilata*	1997	1997						1														1
*Chaetomium perlucidum*		2003	2003		1					1		1											3
***Cladosporium***				**1**	**4**	**4**		**2**	**7**	**7**			**1**							**17**			**43**
*Cladosporium bruhnei*	*Cladosporium allicinum*	1994	2014						1				1							1			3
*Cladosporium castellanii*		2005	2005																	1			1
*Cladosporium cladosporioides*		1975	2019		1	3		1	3	6										9			23
*Cladosporium herbarum*		1994	2012						3											1			4
*Cladosporium langeronii*		2018	2018																	1			1
*Cladosporium macrocarpum*		2011	2011		1																		1
*Cladosporium oxysporum*		1999	2006																	2			2
*Cladosporium sphaerospermum*		2003	2019	1	2	1		1		1										2			8
***Ochroconis***				**1**	**2**	**1**				**5**										**6**	**1**		**16**
*Ochroconis constricta*		2014	2014	(1)	(2)	(1)				(3)										1	(1)		1 (9)
*Dactylaria constricta*	*Ochroconis constricta*	1992	2012	1	2	1				3											1		8
*Ochroconis cordanae*		2014	2014																	1			1
*Ochroconis mirabilis*		2014	2016							1										2			3
*Ochroconis musae*		2018	2018																	1			1
*Ochroconis olivacea*		2014	2014							1													1
*Ochroconis tshawytschae*		2012	2012																	1			1
***Phaeoacremonium***				**4**	**2**	**3**				**3**		**1**					**6**	**1**	**1**	**29**			**50**
*Phaeoacremonium aleophilum*		2003	2011																	2			2
*Phaeoacremonium fuscum*		2015	2015																	1			1
*Phaeoacremonium inflatipes*		1998	2014	1		1														2			4
*Phaeoacremonium krajdenii*		2006	2006																	1			1
*Phaeoacremonium parasiticum (Phialophora parasitica)*		1983	2019	3	2	2				3		1					5	1	1	18			36
*Phaeoacremonium rubrigenum*		1999	2012																	3			3
*Phaeoacremonium sphinctrophorum*		2016	2016																	1			1
*Phaeoacremonium venezuelense*		2006	2012														1			1			2
***Rhinocladiella***					**15**	**1**	**2**							**1**			**1**			**8**			**28**
*Rhinocladiella aquaspersa*		1983	2019				2													4			6
*Rhinocladiella atrovirens*		1989	1989		1																		1
*Rhinocladiella basitona*		2013	2015			1														1			2
*Rhinocladiella mackenziei*		2009	2020		14									1			1						16
*Rhinocladiella similis*		2017	2020																	3			3
***Fonsecaea***				**1**	**9**	**6**	**3**		**4**	**6**				**1**			**2**		**1**	**147**			**180**
*Fonsecaea monomorpha*		2005	2019		2	1														10			13
*Fonsecaea nubica*		2010	2020				1													14			15
*Fonsecaea pedrosoi*		1973	2020	1	7	5	2		4	5 (6)				1			2		1	107 (122)			135 (151)
*Fonsecaea compactum*	*Fonsecaea pedrosoi*	1983	1989																	2			2
*Fonsecaea compactum/compacta*	*Fonsecaea pedrosoi*	1983	2007																	5			5
*Hormodendrum pedrosoi*	*Fonsecaea pedrosoi*	1961	1978							1										2			3
*Phialophora pedrosoi*	*Fonsecaea pedrosoi*	1951	1994																	6			6
*Fonsecaea pugnacius*		2015	2015																	1			1
***Phialophora***						**8**	**1**		**2**		**1**	**1**					**7**		**1**	**39**			**60**
*Phialophora americana*		2019	2019																	1			1
*Phialophora hoffmannii*		1982	1982																	1			1
*Phialophora reptans*		2011	2011																	1			1
*Phialophora richardsiae*		1968	2004			1						1					5		1	11			19
*Phialophora verrucosa*		1968	2019			7	1		2		1						2			25			38
***Phoma***						**1**				**1**										**5**			**7**
*Phoma exigua*		2006	2006							1													1
*Phoma glomerata*		2008	2008			1																	1
*Phoma herbarum*		2010	2010																	1 (2)			1 (2)
*Phoma hibernica*	*Phoma herbarum*	1970	1970																	1			1
*Phoma minutella*		1987	1987																	1			1
*Phoma minutispora*	*Westerdykella minutispora*	1984	1984																	1			1
*Phoma sorghina*		1989	1989																	1			1
***Madurella***					**1**				**2**	**1**			**1**				**15**	**4**		**94**			**118**
*Madurella fahalii*		2012	2014																	2			2
*Madurella mycetomatis*		1985	2020		1				(2)	1			1				13 (15)	4		63 (88)			83 (112)
*Madurella mycetomi*	*Madurella mycetomatis*	1956	2013						2								2			25			29
*Madurella pseudomycetomatis*		2010	2020																	3			3
*Madurella tropicana*		2012	2012																	1			1
***Other Genus***				**21**	**16**	**42**	**1**	**1**	**20**	**33**		**10**	**14**	**2**	**5**		**10**	**1**	**3**	**200**			**379**
*Achaetomium strumarium*		2018	2018																	1			1
*Acrophialophora fusispora*		1983	2020		3	5			1	5													14
*Acrophialophora levis*		2015	2019		1					2										1			4
*Anthopsis deltoidea*		1984	1984														1						1
*Arnium leporinum*		1984	1984									1			1		1						3
*Arthrinium arundinis*		2017	2018																	4			4
*Arthrinium phaeospermum*		1989	1989																	1			1
*Ascotricha chartarum*		1996	2019						1	1													2
*Aureobasidium melanogenum*		2016	2016																	1			1
*Aureobasidium proteae*		2012	2012		1																		1
*Aureobasidium pullulans*		1971	2019	11		5		1	1	3			6		1					13			41
*Cladorrhinum bulbillosum*		2011	2011			1																	1
*Cochliobolus hawaiiensis*		2015	2015						1														1
*Lecythophora hoffmannii*	*Coniochaeta hoffmannii*	1997	1997						1														1
*Phialophora mutabilis*	*Coniochaeta mutabilis*	1973	1991			1						2											3
*Coniochaeta polymorpha*		2013	2013						1														1
*Coniothyrium fuckelii*		1987	1987											1									1
*Corynespora cassiicola*		1969	2019			1														8			9
*Cyphellophora pluriseptata*		1986	2002																	2			2
*Pseudomicrodochium fusarioides*	*Cyphellophora fusarioides*	1991	1991							1													1
*Drechslera dematioidea*		2005	2005																	1			1
*Drechslera rostrata*		1986	1986																	1			1
*Dichotomophthoropsis nymphaearum*		1990	1990			1																	1
*Hormonema dematioides*		1990	1998	1									1							1			3
*Hortaea werneckii*		2005	2019	1		(1)							1							13 (26)			15 (29)
*Cladosporium werneckii*	*Hortaea werneckii*	1964	1978																	6			6
*Exophiala werneckii*	*Hortaea werneckii*	1980	2000			1														7			8
*Lasiodiplodia theobromae*		1976	2019	1		12			4	1										3			21
*Lecythophora mutabilis*		1985	2011	1		4						2	1							1			9
*Leptosphaeria senegalensis*		1960	2006																	4			4
*Macrophomina phaseolina*		2008	2020			2											1		1	1			5
*Microsphaeropsis arundinis*		2004	2019																2	5			7
*Microsphaeropsis olivacea*		1999	2001			1														1			2
*Mycoleptodiscus indicus*		1995	2012														2	1		3			6
*Neoscytalidium dimidiatum*		2009	2019	(2)	(2)	(1)			1 (4)	1 (2)										17 (46)			19 (57)
*Scytalidium dimidiatum*	*Neoscytalidium dimidiatum*	1993	2015	2	2	1			3	1										29			38
*Neotestudina rosatii*		1968	1982																	3			3
*Nigrospora oryzae*		2014	2014																	1			1
*Nigrospora sphaerica*		2009	2020			1														2			3
*Oidiodendron cerealis*		1969	1969																	1			1
*Papulaspora equi*		2014	2020			2																	2
*Phaeosclera dematioides*		1987	1996						1											3			4
*Phomopsis bougainvilleicola*		2013	2013														1						1
*Phomopsis longicolla*		2011	2011																	1			1
*Piedraia hortae*		1978	1997																	4			4
*Pleurophomopsis lignicola*		1995	2004						1											3			4
*Phialophora repens*	*Pleurostoma repens*	1975	1996																	3			3
*Pleurostomophora richardsiae*		2012	2019	1					1											4			6
*Pseudochaetosphaeronema larense*		1987	2014																	3			3
*Pseudochaetosphaeronema martinelli*		2015	2015																	1			1
*Phoma cava*	*Pyrenochaeta cava*	1997	1997																	1			1
*Pyrenochaeta unguis-hominis*		1980	2020																	3			3
*Scytalidium cuboideum*		2013	2013						1	1													2
*Scytalidium hyalinum*		1977	2018			1														21			22
*Scytalidium lignicola*		1983	2020			1														4			5
*Sphaeropsis subglobosa*		1991	1991			1																	1
*Tetraploa aristata*		1990	2013							1										1			2
*Thermomyces lanuginosus*		1991	1991									1											1
*Thermothelomyces thermophila*		2017	2017		1					1		1	1		1		1			1			7
*Myceliophthora thermophila*	*Thermothelomyces thermophila*	1992	2011	2	1					3		3	1		1		1			1			13
*Ulocladium botrytis*		2004	2010						1											1			2
*Ulocladium chartarum*		1981	2003																	2			2
*Veronaea botryosa*		2003	2018			0														10			10
*Verruconis gallopava*		2014	2020	(1)	1 (7)	(1)	1		1	6 (12)			(3)	(1)	(1)		(2)			1 (5)			10 (35)
*Dactylaria gallopava*	*Verruconis gallopava*	1990	2001		4					2			1	1	1					1			10
*Ochroconis gallopava*	*Verruconis gallopava*	1986	2018	1	2	1				4			2				2			3			15
***Scedosporium/Lomentospora* complex**				**98**	**126**	**132**	**27**	**1**	**73**	**253**	**1**	**50**	**35**	**17**	**44**	**3**	**110**	**4**	**21**	**211**	**16**		**1222**
*Lomentospora prolificans*		2015	2020	7 (52)	4 (21)	4 (17)	(2)	(1)	(11)	10 (56)		1 (17)	(13)	(8)	(15)		4 (25)	(1)	2	2 (18)	(2)		34 (261)
*Scedosporium prolificans*	*Lomentospora prolificans*	1993	2017	45	17	13	2	1	11	46		16	13	8	15		21	1		16	2		227
*Scedosporium apiospermum*		1981	2020	18	40 (43)	56 (64)	15 (16)		25 (27)	86 (94)	1	13	7	3	6 (8)	1	42 (45)	1	14	96 (110)	3		427 (468)
*Monosporium apiospermum*	*Scedosporium apiospermum*	1953	1993		3	8	1		2	8					2		3			12			39
*Polycytella hominis*	*Scedosporium apiospermum*	1987	2006																	2			2
*Scedosporium aurantiacum*		2005	2019	2	1	3	3		1	14							2			3			29
*Scedosporium boydii*		2014	2020	(13)	1 (55)	2 (44)	(5)		(29)	4 (79)		1 (15)	(5)	(2)	(14)	(2)	(33)		(2)	2 (72)	(5)		10 (375)
*Allescheria boydii*	*Scedosporium boydii*	1948	1996		8	9			2	17		2			1		4			12	2		57
*Petriellidium boydii*	*Scedosporium boydii*	1976	1997	1	7	6	1		5	9		3			1		5			10			48
*Pseudallescheria angusta*	*Scedosporium boydii*	2011	2019							1										1			2
*Pseudallescheria boydii*	*Scedosporium boydii*	1982	2017	12	39	27	4		22	48		9	5	2	12	2	24		2	47	3		258
*Scedosporium dehoogii*		2017	2018																	2			2
*Scedosporium inflatum*		1990	2012	13	6	4	1		5	9		5	10	4	7		5	2	3	6	6		86
*Pseudallescheria minutispora*	*Scedosporium minutisporum*	2013	2013							1													1
**Others**				**66**	**31**	**150**	**5**		**104**	**140**	**2**	**22**	**52**	**10**	**7**	**1**	**16**	**4**	**5**	**254**	**2**		**871**
*Acremonium atrogriseum*		2000	2000			1																	1
*Acremonium implicatum*		2001	2012			1														1			2
*Acremonium potronii*		1975	2015			1														1			2
*Acremonium sclerotigenum*		2011	2014	1																2			3
*Albifimbria verrucaria*		2020	2020	1		1																	2
*Amesia atrobrunnea*		2019	2019																	1			1
*Amylomyces rouxii*		2018	2018						1														1
*Aphanoascus fulvescens*		1970	1992																	3			3
*Arachnomyces kanei*		2002	2002																	1			1
*Arthrobotrys oligospora*		1990	1990			1																	1
*Arthrographis kalrae*		1997	2020	3	2	4			3	3		1					2			3			21
*Arthropsis hispanica*		2013	2013							1										1			2
*Auxarthron ostraviense*		2013	2013																	1			1
*Basidiobolus haptosporus*		1978	2020						2				3					1		8			14
*Beauveria bassiana*		1997	2016			8			1	2										2			13
*Botryosphaeria dothidea*		2020	2020																	2			2
*Carpoligna pleurothecii*		2010	2010			1																	1
*Cephaliophora irregularis*		1995	1995			1																	1
*Cephalotheca foveolata*		2006	2006																	1			1
*Ceratocystis adiposa*		2014	2014						1														1
*Cercospora apii*		1957	1957																	1			1
*Chalara ellisii*		1999	1999														1						1
*Neurospora sitophila*	*Chrysonilia sitophila (Asexual State)*	1997	1997							1													1
*Chrysosporium articulatum*		2015	2015							1													1
*Chrysosporium georgii*		2001	2001																	1			1
*Chrysosporium keratinophilum*		2017	2017																	1			1
*Chrysosporium parvum*		1973	2007	1		1				7							1			2			12
*Chrysosporium tropicum*		2007	2007						1														1
*Chrysosporium zonatum*		1999	2016							3							1						4
*Colletotrichum coccodes*		2015	2015			1																	1
*Colletotrichum crassipes*		2001	2001																	1			1
*Colletotrichum dematium*		2004	2019			7																	7
*Colletotrichum gloeosporioides*		1998	2020			10 (11)													1	4			15 (16)
*Glomerella cingulata*	*Colletotrichum gloeosporioides*	1983	1983			1																	1
*Colletotrichum siamense*		2019	2019																	1			1
*Colletotrichum truncatum*		2011	2020			3														1			4
*Conidiobolus coronatus*		1978	2021	3	1				47 (57)	2		1			1	1				3	1		60 (70)
*Entomophthora coronata*	*Conidiobolus coronatus*	1965	2006						10														10
*Conidiobolus incongruus*		1983	2010	1					4	2		1	1	1						3			13
*Conidiobolus lamprauges*		2011	2011							1			1	1	1								4
*Coniosporium epidermidis*		2008	2012																	3			3
*Paecilomyces javanicus*	*Cordyceps javanica (Teleomorph)*	1984	1986	1	1							2											4
*Paecilomyces farinosus*	*Cordyceps farinosa*	1994	1994			1																	1
*Thielavia sepedonium*	*Corynascus sepedonium*	1990	1990																	1			1
*Cryptendoxyla hypophloia*		2014	2014																	1			1
*Cryptostroma corticale*		1962	1966							2													2
*Cylindrocarpon destructans*		1991	2011																	2			2
*Cylindrocarpon lichenicola*		1997	2012			5				1			1							5			12
*Daldinia eschscholtzii*		2015	2015																	1			1
*Diaporthe phaseolorum*		2011	2019																1	2 (3)			3 (4)
*Phomopsis phaseoli*	*Diaporthe phaseolorum (Teleomorph)*	2011	2011																	1			1
*Didymella microchlamydospora*		2019	2019							1													1
*Edenia gomezpompae*		2013	2013			1																	1
*Emarellia grisea*		2016	2016																	1			1
*Emarellia paragrisea*		2016	2016																	1			1
*Emergomyces africanus*		2017	2019	2																3			5
*Emergomyces canadensis*		2018	2018	1						1										1			3
*Emergomyces orientalis*		2017	2017							1										1			2
*Emergomyces pasteurianus*		2015	2020	1						2	1									5			9
*Emmonsia crescens*		1964	2012	1					1	17			2							1			22
*Engyodontium album*		1983	2016		1	1 (2)						2								1			5 (6)
*Beauveria alba*	*Engyodontium album*	1984	1984			1																	1
*Epicoccum nigrum*		1997	2020			1			2														3
*Setosphaeria holmii*	*Exserohilum holmii*	2018	2018			1																	1
*Falciformispora senegalensis*		2014	2014																	1			1
*Fuscoporia ferrea*		2010	2010							1													1
*Geomyces pannorum*		2003	2008																	3			3
*Cephalosporium serrae*	*Gibellulopsis serrae*	1974	1974																	1			1
*Acremonium polychromum*	*Gliomastix polychroma*	2004	2004						1														1
*Graphium basitruncatum*		2007	2017	2																1			3
*Gymnascella dankaliensis*		1989	2007																	2			2
*Hongkongmyces pedis*		2014	2014																	1			1
*Hormographiella aspergillata*		1996	2019		3	2			1	11			1							3			21
*Hypocrea orientalis*		2008	2008										1										1
*Inonotus (Phellinus) tropicalis*		2005	2017	1						1							1			2			5
*Isaria farinosa*		2013	2013										1 (2)										1 (2)
*Paecilomyces fumosoroseus*	*Isaria fumosorosea*	2015	2015										1										1
*Knufia epidermidis*		2019	2019																	1			1
*Laetisaria arvalis*		2018	2018			1																	1
*Botryodiplodia theobromae*	*Lasiodiplodia theobromae*	1975	1976			1														1			2
*Malbranchea pulchella*		1951	1994						1											1			2
*Medicopsis romeroi*		2016	2019			1											1		1	7			10
*Metarhizium anisopliae*		1997	2017			4														1			5
*Metarhizium pingshaense*		2017	2017							1													1
*Metarhizium robertsii*		2017	2017			1			1														2
*Paecilomyces viridis*	*Metarhizium viride*	1975	1975			1																	1
*Microcyclosporella mali*		2015	2015			1																	1
*Fusarium nivale*	*Microdochium nivale*	1966	1966			1																	1
*Monascus ruber*		2010	2018										1							1			2
*Mycotypha microspora*		2018	2018										1										1
*Myriodontium keratinophilum*		1985	1985						1														1
*Ramichloridium schulzeri*	*Myrmecridium schulzeri*	1985	1985						1														1
*Nannizziopsis obscura*		2017	2017	1														1		1			3
*Nattrassia mangiferae*		1997	2010		3	4 (5)														7 (41)			14 (49)
*Hendersonula toruloidea*	*Nattrassia mangiferae*	1970	2004			1														34			35
*Acremonium falciforme*	*Neocosmospora falciformis*	1976	2019	1		3							2				1			10			17
*Neocucurbitaria keratinophila*		2019	2019			2														1			3
*Nigrograna mackinnonii*		2013	2020																	4			4
*Onychocola canadensis*		1994	2016																	17			17
*Ophiostoma piceae*		2009	2009		1					1													2
*Ovadendron sulphureo-ochraceum*		1995	1995			1																	1
*Paecilomyces formosus*		2016	2017							1										1			2
*Paecilomyces marquandii*		1979	2000																	2			2
*Thermoascus taitungiacus*	*Paecilomyces taitungiacus (Anamorph)*	2001	2001										1										1
*Paecilomyces variotii*		1981	2019	6 (7)	3	4	2		3	10 (12)	1	1 (4)	10 (12)	2	2		1 (2)		(1)	8 (9)			53 (64)
*Paecilomyces varioti*	*Paecilomyces variotii (Orthographic Variant)*	1971	1998	1						2		3	2				1		1	1			11
*Pallidocercospora crystallina*		2019	2019																	1			1
*Parathyridaria percutanea*		2019	2019																	1			1
*Pestalotiopsis clavispora*		2013	2013			1																	1
*Petromyces alliaceus*		2007	2007				1			1													2
*Phaeoisaria clematidis*		2000	2000			1																	1
*Phanerochaete chrysosporium*		2014	2014							1													1
*Phanerochaete sordida*		2017	2017							1													1
*Pleurostoma ootheca*		2014	2014																	1			1
*Pleurostoma richardsiae*		2017	2019	1		1								1			0			2			5
*Podospora austroamericana*		2018	2018			1																	1
*Pseudopestalotiopsis theae*		2019	2019			1																	1
*Pseudotaeniolina globosa*		2003	2003	1																			1
*Purpureocillium lilacinum*		2011	2020	1	1	9			3	3		2								9			28
*Pythium insidiosum*		1993	2021	15	1	36			2	1		1	1	1	1			1	1	4			65
*Ramichloridium obovoideum*	*Ramichloridium makenziei*	1988	1999		3																		3
*Rasamsonia aegroticola*		2015	2017			1				3		1			1			1		1	1		9
*Rasamsonia argillacea*		2010	2018	1	(3)				(1)	5 (13)							(1)			(1)			6 (20)
*Geosmithia argillacea*	*Rasamsonia argillacea*	2010	2015		3				1	8							1			1			14
*Penicillium emersonii*	*Rasamsonia emersonii*	1999	1999							1													1
*Rasamsonia piperina*		2016	2019							1			1				1						3
*Rhizoctonia solani*		2012	2013			1														1			2
*Rhytidhysterium rufulum*		2014	2018																	4			4
*Roussoella solani*		2017	2017			1																	1
*Sarocladium kiliense*		-	-	(5)		(7)			(1)	(2)		(1)	(2)				(2)			(10)			(30)
*Acremonium kiliense*	*Sarocladium kiliense*	1981	2017	5		6			1	2		1	2				2			8			27
*Cephalosporium madurae*	*Sarocladium kiliense*	1962	1966																	2			2
*Cephalosporium niveolanosum*	*Sarocladium kiliense*	1960	1960			1																	1
*Acremonium strictum*	*Sarocladium strictum*	1984	2015	7	2	1				8		2	3	1	1		1			9			35
*Stachybotrys atra*		1999	1999							3													3
*Stachybotrys chlorohalonata*		2021	2021						1														1
*Stachybotrys eucylindrospora*		2014	2014			1																	1
*Thermoascus aurantiacus*		1967	1967							1													1
*Thermoascus crustaceus*		2010	2019							2			4										6
*Thielavia subthermophila*		2009	2011		1	1																	2
*Tilletiopsis minor*		1997	2018						1	1										1			3
*Tintelnotia destructans*		2018	2019			2																	2
*Toxicocladosporium irritans*		2011	2011																	1			1
*Triadelphia pulvinata*		2001	2013	1																1			2
*Trichoderma bissettii*		2014	2014						1	1							1			1			4
*Trichoderma citrinoviride*		1999	2008	1						1													2
*Trichoderma harzianum*		1996	2014	1	1	1			2	3			1							1			10
*Trichoderma longibrachiatum*		1995	2019	2	3		2		6	9		4	7	2						5			40
*Trichoderma orientale*		2014	2014	1					1	1			1							1			5
*Trichoderma pseudokoningii*		1995	2000		1					1			1							1			4
*Trichoderma viride*		1976	2005							2			2	1									5
*Tritirachium oryzae*		2010	2018																	4			4
*Tritirachium roseum*		1975	1975			1																	1
*Truncatella angustata*		2015	2015																	1			1
*Stemphylium lanuginosum*	*Ulocladium lanuginosum*	1983	1983							1													1
*Ustilago echinata*		2016	2016																	1			1
*Ustilago esculenta*		1996	2007							2													2
*Valsa sordida*		2006	2008						2	1													3
*Westerdykella dispersa*		2014	2018	1																1			2
*Xanthothecium peruvianum*		2017	2017																	1			1
*Acremonium recifei*	*Xeneoacremonium recifiei*	1979	2010			2														3			5
*Xylaria enteroleuca*		2006	2006						1														1
Total	*-*	-	-	**629**	**669**	**958**	**259**	**12**	**1046**	**2511**	**14**	**333**	**354**	**118**	**252**	**21**	**474**	**62**	**139**	**2499**	**74**	**1**	**10,435**

CNS: central nervous system; ORL: oto-rhino-laryngological; OA: osteo-articular; and (nb): number of publications corresponding to all isolates from the same species on the basis of their current taxonomic.

**Table 2:** Anatomical sites and nosological framework of the different taxa. CNS: central nervous system; and ORL: oto-rhino-laryngological. The numbers in the table correspond to PMIDs.

	*Aspergillus*	Dematiaceous	*Pseudallescheria/Scedosporium*	Other	Mucorales	*Fusarium*	*Penicillium*	Total
**Systemic**	**193**	**75**	**105**	**56**	**46**	**138**	**54**	**678**
Anatomical site								
Unspecified	19		8		18	2		47
Blood	91	49	78	24	13	128	31	427
Bone marrow	5	1	2	4			13	25
Lymph nodes	17	15	7	6	2	2	10	57
Semiology								
Aortitis	29	6	6	1	1	1		44
Vasculitis	32	4	4	21	12	5		78
**CNS**	**288**	**205**	**133**	**38**	**41**	**8**	**3**	**710**
Anatomical site								
Unspecified	127	39	46	16	25	2	2	255
Specimen								
Brain abscess	79	114	59	13	8	3		273
Semiology								
Encephalitis	1	4	1	1				6
Mass	37	19	3	4	5			67
Meningitis	39	25	17	3	3	3	1	93
Meningo-encephalitis	5	4	7	1				16
**Ocular**	**321**	**168**	**147**	**155**	**20**	**207**	**9**	**1021**
Anatomical site								
Unspecified	42	9	28	7	2	4	1	94
Conjonctival	6	2	3	2				13
Orbital	20	2	8	5	3	1		38
Specimen								
Lacrimal fluid	3		2	1	1			7
Semiology								
Blepharitis	1							1
Endophthalmitis	87	39	43	19	4	41	3	240
Granuloma	5							5
Keratitis	157	116	63	121	10	161	5	623
**Auditory system**	**193**	**17**	**29**	**8**	**13**	**1**	**6**	**263**
Anatomical site								
Unspecified	17	8	10	1	3			38
Semiology								
Implant-associated otomycosis		1	1		1			3
Otomycosis	176	8	18	7	9	1	6	222
**Dental and gums**	**5**	**5**	**1**			**1**	**1**	**13**
Anatomical site								
Unspecified	4	4				1	1	10
Specimen								
Abscess		1	1					2
Semiology								
Peridontitis	1							1
**ORL sphere**	**658**	**153**	**87**	**133**	**179**	**38**	**22**	**1246**
Anatomical site								
Unspecified	5		1	1		1	1	9
Cervical lymphadenopathy	1							1
Laryngeal	18	1			1		2	22
Nasal	81	23	5	11	7	8	4	139
Oesophagus	7	3	3	1	1	4	2	22
Oral mucosa	20	7	2	3	5	2		39
Rhinitis		1						1
Rhino-facial	1	3	1	47	9			60
Rhino-orbital	51	8	6	11	33			105
Rhino-orbito-cerebral	20		1	9	64			89
Rhino-sinusal	282	87	51	38	41	17	8	514
Sino-oral	11	3		3	8	1		26
Tongue	7				1			9
Tonsil		1						1
Tracheal	103	6	10	5	9	3	1	135
Semiology								
Fungus ball	44	8	7	4		2	1	62
Pharyngeal abscess	3							3
Pharyngitis	4	2					3	9
**Pulmonary**	**3088**	**238**	**424**	**218**	**292**	**51**	**65**	**4353**
Anatomical site								
Unspecified	208	13	25	13	21	9		287
Lower respiratory tract	999	100	166	93	121	20	22	1509
Lymph nodes	84	13	8	13	21	1	2	141
Mediastinum	37	2	2	3	4			48
Parenchymal cavity	331	12	46	8	28	1	6	430
PleurisyPleura	74	11	13	8	11	1	5	125
Upper respiratory tract	694	48	111	34	31	8	12	934
Specimen								
Abscess	40	3	13	6	2	1		63
Semiology								
Hypersensitivity/Allergy	106	8		3		1	3	120
Invasive	389	9	12	14	28	1	9	462
Pneumonia	126	19	28	23	25	8	6	234
**Breast**	**7**	**3**	**1**	**2**	**1**	**1**	**1**	**16**
Anatomical site								
Unspecified	1	3		1	1	1		7
Breast implant	2		1	1				4
Nipples	1							1
Specimen								
Milk	3						1	4
**Heart**	**219**	**31**	**54**	**24**	**29**	**13**	**3**	**372**
Anatomical site								
Unspecified	37	4	11	1	16	4		74
Myocardium	30	3	9	2	4	2		49
Pericardium	23	3	3	5	2		2	37
Thrombus	8		2		1			11
Semiology								
Implanted device endocarditis	55	14	12	10		2	1	96
Native valve endocarditis	66	7	17	6	6	5		105
**Digestive**	**149**	**49**	**43**	**54**	**94**	**22**	**12**	**422**
Anatomical site								
Unspecified	9	2	4	4	13	1	3	36
Appendix	1			2	3			6
Biliary tract	6	3	2					11
Bowel	14	1	3	5	9	1	3	36
Gastric	10	1	1	5	15	1		34
Pancreas	7		3		3	1		14
Peritoneum	43	32	7	27	16	8	1	135
Spleen	33	5	15	3	18	6	1	80
Specimen								
Abscess	2		1	1	1			5
Faecal specimen	15	4	5	6	6	3	4	43
Semiology								
Colitis	2		2		1			5
Enteritis	2			1	6			9
Mucosal necrosis	5	1			3	1		10
**Liver**	**43**	**10**	**17**	**11**	**34**	**4**	**2**	**120**
Anatomical site								
Unspecified	27	8	17	10	23	3	2	89
Specimen								
Abscess	11	1		1	11	0		24
Ascites fluid	3	1				1		5
Semiology								
Hepatitis	2							2
**Urinary tract**	**159**	**16**	**51**	**14**	**61**	**16**	**1**	**314**
Anatomical site								
Unspecified	3							3
Bladder	6	1			4			11
Kidney	86	10	30	9	46	7	1	185
Prostate gland	7	1	1		1			10
Specimen								
Urine	28	3	13	2	7	8		62
Semiology								
Mass	26		2		1			29
Pyelonephritis	2	1	2	3	2			9
Urinary tract infection	1		3			1		5
**Genital**	**9**	**1**	**3**	**1**	**6**	**1**		**21**
Anatomical site								
Unspecified	3							3
Epididymis		1						1
External genitalia			3		1			4
Glans	1				3	1		5
Ovaries					1			1
Testis	4							4
Vaginal mucosa	1			1	1			3
**Osteo-articular system**	**287**	**84**	**151**	**22**	**28**	**19**	**4**	**589**
Anatomical site								
Unspecified	38	9	18	4	9	2		79
Joint	26	8	21	1	4	1		60
Specimen								
Synovial fluid	8	13	20	1	1	4		46
Semiology								
Arthritis	6	13	28	3	2	6		57
Bursitis		1						1
Mass (including mycetoma)	6	3						9
Osteomyelitis	147	35	55	12	11	5	4	266
Spondylodiscitis	56	2	9	1	1	1		71
**Skeletal muscles**	**21**	**8**	**4**	**8**	**23**	**1**		**61**
Anatomical site								
Unspecified	21	8	4	8	23	1		61
**Soft-tissue**	**44**	**13**	**21**	**12**	**50**	**4**		**137**
Anatomical site								
Unspecified	44	13	21	12	50	4		137
**Skin system**	**457**	**1154**	**239**	**361**	**376**	**270**	**23**	**2801**
Anatomical site								
Unspecified	43	76	17	5	26	5		173
Nails	93	156	3	77	3	65	1	394
Subcutaneous	56	343	47	78	84	25		610
Superficial cutaneous	215	370	91	149	248	139	21	1195
Semiology								
Dermatitis	1	2		1	1	1	1	7
Intertrigos						1		1
Mycetoma	15	155	64	46		16		282
Tinea capitis	1	1						2
Tinea corporis		1		3				4
Tinea manuum		3						3
Tinea pedis		8		1				9
Ulcer	33	39	17	1	14	18		121
**Endocrine gland**	**40**	**7**	**18**	**4**	**15**	**2**		**84**
Anatomical site								
Unspecified		1	2					3
Adrenal	7	2			3	1		13
Parathyroid	1							1
Thymus					1			1
Thyroid	32	4	16	4	11	1		66
**Placental infection**	**1**							**1**
Anatomical site								
Placenta	1							1
**Total**	6182	2237	1528	1121	1308	797	206	13,222

**Table 3:** Details of cardiac sites affected by native valve endocarditis and associated species.

Current Name	Tricuspid Valve	Mitral Valve	Aortic Valve	All 4 Chambers	Mural Endocardiumm	Pulmonary Valve	Atrium	Ventricle	ND
*Absidia corymbifera*	1 [65]								
*Arnium leporinum*		1 [66]							
*Arthrographis kalrae*			1 [67]						
*Aspergillus clavatus*			1 [68]						
*Aspergillus flavus*	6 [69,70,71,72,73,74]	9 [69,73,75,76,77,78,79,80,81]	2 [74,82]						
*Aspergillus fumigatus*	4 [83,84,85,86]	18 [87,88,89,90,91,92,93,94,95,96,97,98,99,100,101,102,103,104]	10 [100,105,106,107,108,109,110,111,112,113]	2 [114,115]	2 [116,117]	2 [116,118]	3 [119,120,121]		2 [122,123]
*Aspergillus nidulans*							1 [124]		
*Aspergillus niger*		1 [125]							
*Aspergillus terreus*	1 [126]	2 [126,127]	1 [128]	1 [126]			1 [127]		1 [129]
*Aspergillus udagawae*		1 [130]							
*Cunninghamella bertholletiae*			1 [131]		1 [132]				
*Engyodontium album*			1 [133]						
*Exophiala dermatitidis*			1 [134]						
*Fusarium incarnatum*									1 [135]
*Fusarium solani*		1 [136]	1 [137]			1 [138]			
*Fusarium solani complex*								1 [139]	
*Lomentospora prolificans*			1 [140]						
*Myceliophthora thermophila*							1 [141]		1 [142]
*Paecilomyces javanicus*			1 [143]						
*Phaeoacremonium parasiticum*	1 [144]	1 [144]	1 [144]						
*Phialemonium obovatum*	1 [145]								1 [146]
*Pseudallescheria boydii*	1 [147]	3 [148,149,150]				1 [151]			
*Saksenaea vasiformis*		1 [152]							
*Scedosporium apiospermum*		1 [153]							
*Scedosporium boydii*									1 [154]
*Scedosporium inflatum*									
*Scedosporium prolificans*	1 [155]	2 [156,157]	2 [158,159]						1 [160]
*Trichoderma longibrachiatum*							1 [161]		
Total	16	41	24	3	3	4	7	1	8

ND: not determined.

## Data Availability

Not applicable.
